# Work environment risk factors causing day-to-day stress in occupational settings: a systematic review

**DOI:** 10.1186/s12889-021-12354-8

**Published:** 2022-02-05

**Authors:** Junoš Lukan, Larissa Bolliger, Nele S. Pauwels, Mitja Luštrek, Dirk De Bacquer, Els Clays

**Affiliations:** 1 Department of Intelligent Systems, Jožef Stefan Institute, Jamova cesta 39, Ljubljana, 1000 Slovenia; 2 Jožef Stefan Postgraduate School, Jamova cesta 39, Ljubljana, 1000 Slovenia; 3 Department of Public Health and Primary Care, Ghent University, C. Heymanslaan 10, Ghent, 9000 Belgium; 4 Knowledge Centre for Health Ghent, Ghent University, C. Heymanslaan 10, Ghent, 9000 Belgium; 5 Ghent University Hospital, C. Heymanslaan 10, Ghent, 9000 Belgium

**Keywords:** Day-to-day stress, Ecological Momentary Assessment (EMA), Work environment risk factors, Stress outcomes, Systematic literature review

## Abstract

**Background:**

While chronic workplace stress is known to be associated with health-related outcomes like mental and cardiovascular diseases, research about day-to-day occupational stress is limited. This systematic review includes studies assessing stress exposures as work environment risk factors and stress outcomes, measured via self-perceived questionnaires and physiological stress detection. These measures needed to be assessed repeatedly or continuously via Ecological Momentary Assessment (EMA) or similar methods carried out in real-world work environments, to be included in this review. The objective was to identify work environment risk factors causing day-to-day stress.

**Methods:**

The search strategies were applied in seven databases resulting in 11833 records after deduplication, of which 41 studies were included in a qualitative synthesis. Associations were evaluated by correlational analyses.

**Results:**

The most commonly measured work environment risk factor was work intensity, while stress was most often framed as an affective response. Measures from these two dimensions were also most frequently correlated with each other and most of their correlation coefficients were statistically significant, making work intensity a major risk factor for day-to-day workplace stress.

**Conclusions:**

This review reveals a diversity in methodological approaches in data collection and data analysis. More studies combining self-perceived stress exposures and outcomes with physiological measures are warranted.

**Supplementary Information:**

The online version contains supplementary material available at (10.1186/s12889-021-12354-8).

## Background

Over the past decades, substantial attention has been directed to research focusing on chronic exposure to stressors in occupational settings and the adverse impact of stress on chronic disease outcomes [1, 2]. Psychosocial risk factors have been the last ones to be considered, but are now widely accepted to be as important as other factors like biological or chemical risks [3]. The influence on mental and cardiovascular health in particular has been confirmed and explained through frameworks at the forefront of stress research, such as the Job Demand-Control-Support model [4–6], the Effort-Reward Imbalance model [7], and the Job Demands-Resources model [8].

Evidence of chronic stressors influencing workers’ health and well-being is accumulating, and several systematic reviews and meta-analyses with a focus on studies investigating such relations are available. There is evidence that psychosocial stress is associated with cardiovascular morbidity and mortality [1], musculoskeletal disorders [9], mental health problems, such as depression and anxiety [2], and health risk behaviour, such as cigarette smoking, alcohol consumption, and overweight [10]. The most commonly studied workplace risk factors include job demand and job control (such as in the Job Demand-Control-Support model) [1], but several others have been studied, such as job insecurity, procedural (in)justice, workplace conflict or bullying [2], and workplace violence [11]. A lot of these risk factors are structural and as such measured at a single time point. On the other hand, a preliminary literature search revealed little information about how these structural risk factors manifest in daily work life and what the specific (if any) work environment risk factors causing day-to-day (i.e., non-chronic) stress are.

Understanding how day-to-day work situations lead to the experience of stress is important for several reasons. Stress measurement often relies on self-reports, which are subject to memory bias [12] and there are indications that chronic assessments are not simply the sum of multiple moment-to-moment ratings [13]. When stress is measured several times a day, these repeated measurements can instead capture stressful situations during or soon after they occur and the risk of memory bias is reduced. This relationship would also be important to understand since, in order to test hypotheses about how particular stressors lead to health outcomes, temporal relationships need to be explored [14, 15]. Finally, the question of how to design stress management interventions is still open [16]. Broad constructs such as work demands and decision latitude are relatively stable aspects of a job and translating the findings from chronic stress research into stress management strategies applicable in every-day working life would require a better understanding of their day-to-day manifestations. To the best of our knowledge, no systematic review with a focus on day-to-day stress and related work environment risk factors has been performed so far.

We were interested in how various situations at work translate to an experience of stress and which situations are the most important for this experience. We named this relationship ‘day-to-day stress’, which differs from chronic stress in that it can entail daily situations in addition to structural characteristics of the workplace. Day-to-day stressors do not necessarily have long-lasting consequences but do influence the perception of work environment and elicit some kind of response from a person. We do not presume any conceptual difference between day-to-day stress and chronic stress or stressors, but rather differentiate them based on the methodology of stress measurement. As such, we did not restrain our selection of studies to review to any particular definition of (day-to-day or chronic) stress. To understand how exposures to work environment risk factors are related to daily variations of stress, we instead focused on the studies that measure these repeatedly with self-reports or continuously using physiological measurements and in real-world occupational settings.

The objective of this systematic review was to explore the onset of day-to-day stress by summarising evidence on potential day-to-day work environment risk factors (stressors), which have an immediate effect on self-perceived stress levels or physiological stress responses, and which may or may not cause chronic stress.

## Materials and methods

We conducted this systematic review by following the Cochrane Handbook for Systematic Reviews [17] and the PRISMA 2009 Checklist [18] and registered it on PROSPERO under the ID: CRD42018105355 [19].

### Eligibility criteria

We first settled on working definitions of the concepts of interest. Throughout this systematic review, we use the term work environment risk factors for stressors, which we defined more specifically as *causes or predictors of stress, potentially occurring on a day-to-day basis within occupational settings*. Furthermore, we defined self-perceived stress outcomes as *consequences of such work environment risk factors which were measured with self-perception-based scales, questionnaires, or surveys*.

As the main eligibility criterion, the studies needed to include work environment risk factors and either self-perceived stress outcomes or physiological stress detection or both. We looked for any (objective) descriptions of work situations and their consequences in terms of stress. We did not constrain the outcomes to any particular definition of stress as long as the authors of the original studies framed them as somehow related to the phenomenon of psychosocial stress.

Both, risk factors and outcomes, were required to be measured repeatedly, so methods capable of producing repeated or continuous measurements had to be used. One such method is Ecological Momentary Assessment [20, EMA], which is a research method allowing participants to report their experiences in real-time and in real-world settings, in which data are collected repeatedly (i.e., more than two measurement points) over time and often through a digital platform such as a smartphone application [21]. Moreover, the phenomenon of interest was day-to-day stress, so studies focusing on chronic stress only were not considered.

We focused on studies set in a real-world working environment, and either the workplace setting or the occupational profile needed to be extractable. Healthy full-time and part-time workers of working age were chosen as the population of interest. Observational quantitative and mixed-methods studies (where only the quantitative part of the latter was of relevance) including at least two measurement points were included.

### Strategies for database searching

We devised a search strategy according to the eligibility criteria described in the previous subsection. The main blocks of the search strategy require that 1) a study deals with stress (or synonymous concepts), which should furthermore be 2) day-to-day or episodic (or similar). We also set 3) the requirements for methods, which could be either ecological momentary assessments or other methods capable of producing repeated measures, and 4) that the setting of interest is the work setting. The full search strategies with indexing terms and free text words for all the databases can be found in Supplementary Figs. 1 to 7 [see Additional file 3].

We evaluated the search strategy with the PRESS checklist [22] before we applied it in the following databases: PubMed, Embase, Web of Science, Scopus, CINAHL, ERIC, and PsycArticles. We first carried out a search on 31 August 2018 and later did an update of the initial search on 3 July 2020. The only limitation we used at the time of the searches was “English language”.

### Study selection process

The studies were selected based on the process described in the PRISMA statement [18]. After merging all the results and manually deduplicating them, we screened the titles and abstracts, and evaluated the full text of the remaining articles for eligibility.

Both title and abstract screening and full-text evaluation were done independently by two authors using the Rayyan software [23]. Conflicts after both screening phases were discussed until consensus was reached. We followed the same procedure when we updated our search.

### Quality assessment strategy

The quality assessment in systematic reviews includes two phases: 1) evaluation of the quality at study level (each study separately) and 2) evaluation of the body of evidence (all included studies together) to give a thorough quality estimate of the evidence at hand.

For the quality assessment at study level, we used the QualSyst tool for quantitative research [24]. This tool offers ‘N/A’ grades for criteria that are inapplicable. Out of the 14 criteria in the tool, we omitted three—random allocation to treatment group (1) and blinding of investigators (2) and subjects (3)—since they are only applicable to intervention studies.

Consequently, each study was assessed according to 11 criteria (e.g., question or objective sufficiently described) on a three point scale. From these, the summary score is calculated, which is a number between 0 and 1, where 1 denotes complete satisfaction of all applicable criteria. This procedure was done independently by two authors and any conflicts were discussed until consensus was reached.

The quality assessment of the body of evidence was evaluated using the GRADE approach [25], including five criteria for downgrading (risk of bias, inconsistency, indirectness, imprecision, and publication bias) and two criteria for upgrading (large magnitude of effect and dose-response gradient) before an overall score was given.

## Results

### Study selection

We applied the search strategy as discussed in Strategies for database searching and retrieved 15362 records. We removed duplicates first and then eliminated irrelevant studies by first screening their titles and abstracts and then considering the full text of a selected subset.

**Fig. 1:**
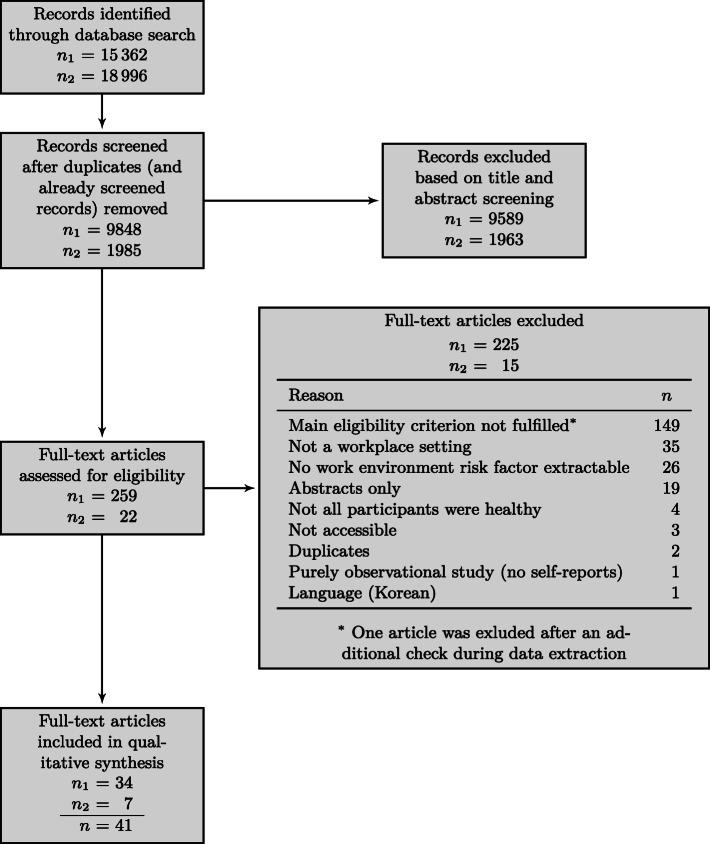
Study selection presented with the PRISMA flow diagram [18]. The *n*_1_ and *n*_2_ refer to the first search we performed on 31 August 2018 and to the update search from 3 July 2020, respectively. Their sum is reported as *n*

We followed the same procedure for the second search, in which we retrieved 18996 records. We excluded all the records already found in the first search as well as some duplicates from different databases, then repeated the same screening procedure. The whole process is illustrated in Fig. 1.

As per recommendations in Moher et al. [18], we noted the exclusion reasons for all the publications we reviewed in full. 149 studies were excluded based on the main eligibility criterion: either the work environment risk factors or stress outcomes were missing, or they were not measured repeatedly. In 35 cases, neither the workplace setting nor the occupational profile was mentioned. In 26 cases, no work environment risk factor could be extracted. 19 publications turned out to be abstracts only. Four further studies were excluded because not all participants were described as healthy. Three studies were not accessible: two PhD theses [26, 27], of which authors were contacted, but we got no response, and a report [28], the author of which is deceased. Two additional duplicates were found, one study only included observational methods (i.e., no self-reports), and one study was available in Korean only.

After this, we were left with 41 studies (34 from the first and 7 from the second search), which were included in the final review for a qualitative synthesis.

### Study characteristics

Table 1 lists all the studies included in the final review and gives their most important characteristics. In addition to the number of subjects included (*N*) and the work setting of the study, it includes the study duration and assessment frequency. Added are the quality rating of the study and information about whether data analysis included multilevel analysis.

**Table 1:** Basic data of all included studies, including the study’s duration (Days), assessment frequency (Freq.), inclusion of multilevel analysis (ML), and the QualSyst score (QS)

ID	Study reference	*N*	Workplace setting	Days	Freq.	ML	QS
1	[48]	83	Hospital nurses	3	1x/d	Yes	0.90
2	[49]	120	Faculty members of a medium-sized university	9	3x/d	No	0.77
3	[50]	304	Nurses (*n*=119) and physicians (*n*=185)	7	1x/90-min	No	0.90
4	[51]	49	Full-time university employees	10	3x/d	Yes	0.86
5	[52]	39	Sales representatives, mechanical engineers, R&D professionals, government service employees, and other	10	1x/d	Yes	0.77
6	[53]	45	Employees of an IT company	15	2x/d	Yes	1.00
7	[54]	40	State police officers	5	1x/d	Yes	0.81
8	[36]	115	Public office employees	5	1x/d	Yes	1.00
9	[55]	106	Employees of public service organizations	5	3x/d	Yes	1.00
10	[56]	14	Emergency response officers (police)	11	1x/d	No	0.85
11	[57]	130	Employees of a security company	28	2x/week	Yes	0.86
12	[58]	205	Employees	5	1x/d	Yes	0.95
13	[59]	64	Professional staff at the headquarters of a construction company	12	1x/d	Yes	1.00
14	[60]	120	Full-time workers	6	–	No	0.90
15	[61]	52	Fly-in–fly-out workers (i.e., workers who fly from cities to remote locations) of a multinational construction company	28	–	Yes	0.86
16	[62]	37	Employees of primary schools	10	1x/d	Yes	0.95
17	[63]	20	Ambulance personnel	7	–	No	0.54
18	[64]	96	Hospital nurses	5	1x/90-min	Yes	0.95
19	[65]	112	Working couples (*n*=56)	7	4x/3-hour	Yes	0.86
20	[66]	76	Service job employees	14	2x/d	Yes	1.00
21	[67]	185	Physicians	7	1x/90-min	No	0.90
22	[68]	133	Hospital nurses	5	4x/d	Yes	0.90
23	[69]	28	Open-floor office workers	42	3x/d	No	0.40
24	[70]	97	Full-time workers	15	1x/d	Yes	0.95
25	[71]	30	Nurses, cooks, salespersons, electronic technicians, bank clerks	15	–	No	0.81
26	[72]		Hospital nurses	60	1x/d	Yes	0.81
	Wave 1:	60		30			
	Wave 2:	38		30			
27	[73]	36	Field emergency medical technicians	30	1x/d	No	0.77
28	[74]	119	Hospital nurses	7	1x/90-min	No	0.95
29	[75]	122	Public office employees	5	2x/d	Yes	0.95
30	[76]						0.50
	Sub-study 1: See [54] (ID 7).						
	Sub-study 2:	41	University secretaries	5	1x/d	Yes	
	Sub-study 3:	38	Correctional officers in prisons	5	1–2x/d	Yes	
31	[77]	20	Surgeons	2–3	–	No	0.70
32	[78]	104	Full-time school teachers	1	–	No	0.63
33	[79]	100	Hospital nurses	2	1x/90-min	Yes	1.00
34	[37]	131	Employees of an IT division	8	1x/d	Yes	1.00
35	[80]	76	Employees (financial and business services, health care, public office, education)	5	3x/d	Yes	1.00
36	[81]	201	Employees	5	1x/d	Yes	0.95
37	[82]	48	Freelance or portfolio workers (publishing, coaching, accountancy, sales, translator, psychologist, web design, joiner)	182	1x/week	Yes	1.00
38	[83]	20	Physicians	1	–	No	0.45
39	[84]	132	Secondary school teachers	3	1x/d	Yes	0.90
40	[85]	23	Hospital nurses	14	–	Yes	0.90
41	[86]	47	Hotel hourly employees (housekeeping, food and beverage, front desk)	8	1x/d	Yes	1.00

The studies included from *N*=14 to *N*=304 participants, with the median *N*_median_=83. The study duration also varied, usually together with the assessment frequency. Thus, some studies only looked at one day, but measured some parameters continuously (e.g., blood pressure in study ID 32), whereas others only sampled once a day for 60 days (study ID 26) or even once a week, but lasted for 182 days (study ID 37).

The average quality of the studies according to QualSyst was *M*_QualSyst_=0.86. Specifically, 10 out of the 41 studies were rated at the highest quality level, 28 studies were rated between 0.99 and 0.51, and 3 studies were rated as 0.50 or below. Points were most often deducted because recruitment methods were not clearly reported (‘Method of subject selection’ criterion) or when the authors only reported significance of the main results, but no estimate of variance, such as confidence intervals or standard errors (‘Some estimate of variance’ criterion).

The GRADE approach rates randomised trials as ‘high quality’, observational studies as ‘low quality’, and any other evidence as ‘very low quality’. Since all included studies of this review are observational studies, the quality of the body of evidence was consequently rated as low. Additionally, according to the criteria for downgrading or upgrading mentioned in Quality assessment strategy, no downgrading was required and no upgrading was feasible for our body of evidence, so ‘low’ was also the final score.

#### Work environment risk factors, self-perceived stress outcomes, and physiological measurements

We identified work environment risk factors, self-perceived stress outcomes, and physiological measurements measured in each study. We extracted all constructs that were measured more than twice (i.e., repeatedly or continuously), while one-time measurements (e.g., during initial baseline screening) were not considered. Since concepts like stress are defined in different ways across the studies, we also looked at the measurement instruments. The work environment risk factors and stress outcomes extracted in this way are published as Supplementary Tables 1 and 2 [see Additional file 1].

We classified these into broader categories, which were based on established frameworks. For work environment risk factors, we used the 6th European Working Conditions Survey classification of job quality indices [29] as the basis. These are objective features of a job which have been proven to have an impact on the health and well-being of workers. They are also only weakly correlated, so that they function as independent descriptions of job quality. Hence, these served well to get a higher level overview of work environment risk factors.

Similarly, stress outcomes were classified according to the stress model described by Ice and James [30]. They describe stress as a combination of affective, behavioural, and physiological responses, which impact mental health and can have physical health outcomes. These responses are consequences of a person’s appraisal of stressors (stimuli). While classifying the studies in our review, we also identified the need for two additional consequences of stress. First, stress can also affect a person’s cognition, not only at the stage of appraisal, but as its consequence; forgetting of intentions and cognitive failure are examples of this outcome. Second, we determined motivational responses, specifically work engagement, to be sufficiently distinct from affective responses to deserve its own category. This outcome involves not only emotions, but goal-directed behaviour closely related to affective responses.

The frequencies of different measures of work environment risk factors and stress outcomes are summarised in Table 2. The risk factor most often measured in the included studies was work intensity, defined for example as time pressure or job demand. This was followed by social environment risk factors (such as co-worker and supervisor support) and ‘various’ factors (such as the number or type of stressful situations). On the other end, affective responses were by far the most often measured outcomes, especially as assessed by the Positive and Negative Affect Scale [31, PANAS]. Some studies looked at physiological responses to stress as well, while other outcomes were rarely considered.

**Table 2:** High-level categories of work environment risk factors and stress outcomes and the corresponding frequencies of measurements of these variables in the included studies (see Tables A1 and A2 in online supplemental material for a complete list of these measures)

Category	Frequency
(a) Work environment risk factors.	
Work intensity	25
Social environment	25
Various	18
Working time quality	10
Skills and discretion	10
Occupation-specific (medicine and health care)	6
Commuting from and to the workplace	4
Prospects	3
Total	101
(b) Stress outcomes.	
Affective response	44
Physiological response	14
Appraisal	5
Behavioural response	4
Motivational response	4
Cognitive outcome	3
Health outcome	2
Total	76

Note that an individual study can include measures of multiple categories

It is important to note that while all studies included at least one work environment risk factor and one stress outcome as a consequence of the design of our eligibility criteria, each study could measure more than one risk factor or outcome. This means that the total number of measurements is larger than the number of included studies.

#### Correlation coefficients

The variables of work environment risk factors, self-perceived stress outcomes, and physiological measurements were considered in the relation structure widely known as ‘exposure/predictor–outcome’ or ‘independent variable–dependent variable’.

As shown in Table 1, 28 studies analysed their data by using multilevel models, while others resorted to other analyses, such as *t*-tests, correlational tables, and descriptive analysis. Since multilevel models control for dependencies between predictors (usually work environment risk factors) they give a more complete insight into relationships between all modelled variables. But for these studies direct comparison of coefficient estimates would not be appropriate since the models’ structure varied from study to study.

To produce a meaningful comparison, we therefore decided to focus on correlation coefficients in our synthesis. As these were available in the 28 studies as well as some others, they enabled us to compare more directly the results of more studies. It needs to be noted, however, that only bivariate relationships are reflected in these analyses, while more complex relationships between variables are omitted.

Accordingly, correlation coefficients between work environment risk factors (exposures) and self-perceived stress outcomes (outcomes), and work environment risk factors (exposures) and physiological measurements (outcomes) are available in Supplementary Table B [see Additional file 2].

For studies that did multilevel analysis, both within-person level and between-persons level results were extracted. But for other studies only a part of the results of interest were extractable. For example, only the results of between-persons level were reported in the study ID 37 or correlation coefficients were not reported for all variables included in the study ID 33 (e.g., no correlation coefficients between nursing tasks and heart rate).

**Fig. 2:**
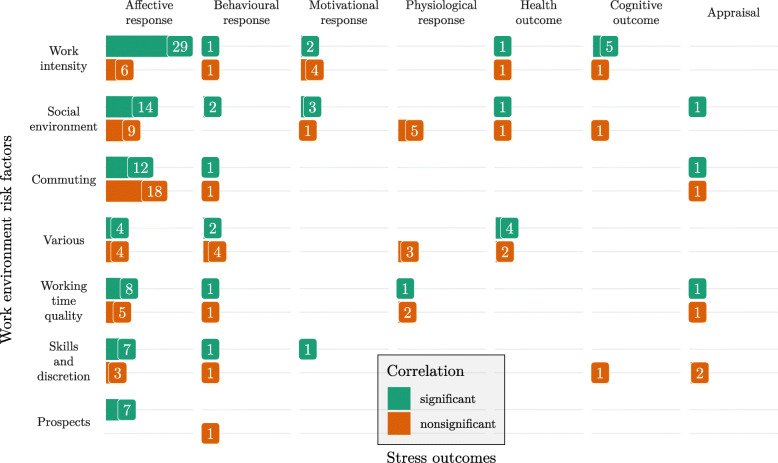
The frequency of significant and nonsignificant correlation coefficients between categories of work environment risk factors and stress outcomes

Figure 2 shows the number of statistically significant and nonsignificant correlation coefficients for each pair of work environment risk factor and stress outcome. In this figure, we focused on within-subject correlations only as these were the ones that can capture day-to-day variation of stressors and responses.

As mentioned in Work environment risk factors, self-perceived stress outcomes, and physiological measurements (see Table 2), the most commonly measured risk factor and outcome were work intensity and affective response, respectively. Correspondingly, their relationship in the form of correlation was also the most commonly reported one. Note that the number of correlation coefficients does not directly follow from the number of studies studying a certain relationship, since Table 2 shows measures of all studies, regardless of whether they did multilevel analysis and whether within-subject correlations could be extracted. Additionally, a study looking at several measures of work environment risk factors or stress outcomes could report a correlation within each pair, so that the number of correlations can be even higher than the number of different measures.

Furthermore, most of the correlations between work intensity and affective response were statistically significant. Affective response was also commonly correlated with social environment and this relationship was more often statistically significant than not.

On the other hand commuting from and to the workplace was not significantly correlated with affective responses most of the time. Interestingly, the second most common type of outcomes, physiological responses, were mostly not significantly correlated with any of the risk factors.

In general, the correlations reported in the included studies were more often significant than not (110 significant vs. 80 nonsignificant correlations), but they were typically low. Only two of all the within-subject correlations exceeded 0.5 in their absolute magnitude.

## Discussion

Among the studies that included within-subject correlation coefficients in their results, the most commonly measured work environment risk factor was work intensity, and it was correlated most often with affective response. The high frequency can be to some extent explained by our search strategy. Both stress and demand were included as search terms and we categorised stress(fulness) as an affective response and work demand as work intensity. But since definitions of stress are diverse and often include concepts such as negative affect, our review captured many other stress responses as well as work environment risk factors (stressors).

Work intensity is often measured in epidemiological studies about health consequences of chronic stress, such as coronary heart disease [32] and depression [33]. This is reflected in the high number of studies that included it as the stressor of interest. However, since work intensity is most often paired with the control dimension to describe job strain, such as in the Job Demand-Control-Support model, the relative rarity of correlations between skills and discretion and stress outcomes is surprising. It seems that the control dimension of this model is relatively less well researched. However, it is still unclear whether demands and control are related to stress and its health consequences independently, or whether their interaction in the form of job strain is more important [34]. To settle this question, it would be crucial to explore the role of the control dimension more carefully.

The social environment (e.g., co-worker and supervisor support) has also been correlated with affective and other responses in the included studies. This should be beneficial for gathering more evidence for the relationship of the support dimension with health outcomes, for which only limited evidence has been available in existing reviews [33]. A similar statement could be made for the prospects category, which has found its place in the Effort-Reward Imbalance model [7].

Another surprising result was that physiological responses were generally not statistically significantly correlated with any of the work environment risk factors, despite the fact that stress is often predicted from physiological parameters [35]. This can be explained either with the studies’ analyses choices or complexity of their models. Some of the studies that measured physiological responses did not perform multilevel analysis, but simpler statistical analyses such as *t*-tests and analysis of variance (studies ID 17, 31, and 32). Others did perform regression analysis or multilevel analysis, but did not report within-subject correlation coefficients for physiological parameters (studies ID 14, 33, and 38). Only three studies reported within-subject correlation coefficients and were included in Fig. 2, but the relationships between work environment risk factors were usually too complex to be captured by these simple coefficients. For example, co-worker support mediated the daily trajectory of some parameters of heart rate variability [36, study ID 8] and the relationship between work-to-family conflict and cortisol slope from dinner to bedtime was mediated by supervisor support [37, study ID 34].

This relative rarity of studies dealing with physiological aspects of stress compared to the field of chronic stress research can be put into a broader context with the help of the conceptual framework proposed by Martikainen et al. [38] and adapted by Rugulies [39]. This framework describes the connection between different levels of work environment and health outcomes. It starts with the broadest, (i) macro-level, economic, social, and political structures, and continues through (ii) meso-level workplace structures, (iii) meso-level psychosocial working conditions, to (iv) individual-level experience and cognitive and emotional processes. The latter elicit either (v) psycho-physiological changes or (vi) health-related behaviours, which in turn impact (vii) workers’ health and illness.

It is well established that meso-level workplace structures (ii), such as job insecurity [40], and meso-level psychosocial working conditions (iii), such as job strain [41], are related to the risk of diseases and disorders (vii). This has been observed both in immediate physiological responses to stress as well as sustained physiological and behaviour changes. For example, longer duration of work-related stress results in increased rise in morning cortisol level and reduced heart rate variability, and acute stress response involves elevated blood pressure [42]. On the other hand, job strain has been found to be linked to hypertension, atherosclerosis, and smoking intensity [43].

Some of the mechanisms of how this happens are also understood. First, pathophysiological effects of stress (v) have been detailed [44], such as neuroendocrine mechanisms of elevated cortisol and catecholamine (epinephrine) levels as well as inhibited anabolism. Second, stress is related to altered behaviour (vi), such as smoking and alcohol consumption [10], where this is seen as a second ‘indirect’ pathway of the link between stress and stress-related diseases.

A causal relationship between stress and cardiovascular diseases has still not been established, however, and the pathological mechanisms of chronic and acute stress may differ [41]. This evidence gap might be owed to a poor understanding of how psychological processes (iv) are involved in this pathway. Steptoe and Kivimäki [41] explicitly limit the focus of their review to ‘exposure to external stressors, rather than on psychological and biological factors affecting vulnerability to adversity’ (p. 360). And while they mention that ‘one reason for the weak relationship between physiological stress responses and future disease is that mental stress testing measures a propensity to high- or low-stress responsivity’ [42, p. 341], they only admit this role to *biological* stress reactivity.

But it might be precisely the psychosocial factors, which Martikainen et al. [38] see as ‘mediating the effects of social structural factors on individual health outcomes’ (p. 1091), that is, the pathway from (ii) and (iii) *through* (iv) to (vii), where the key to better understanding this causal relationship lies. It might be *through* perceptions and psychological processes at the individual level that these macro- and meso-level social processes lead to direct psychobiological processes or modified health-related behaviours and lifestyles and, in turn, influence health [38].

Some of the studies included in our review deal with the relationships in this pathway and more systematic research is needed. This also has implications for planning interventions better, since effects of stress and depression management techniques on cardiac outcomes are still uncertain [41]. While it is clear that occupational stress increases risk for coronary heart disease [45], more research is needed on how to lower this risk. For example, it is possible to modify work schedule to ameliorate exposure to job strain, but only a randomized clinical trial which would test this intervention could truly assess its effect [43].

### Strengths and limitations

As illustrated, different methods of data collection (e.g., time span, number of measurement points) and a wide range of data analysis approaches (e.g., descriptive results, multilevel analysis) were used across the included studies. This heterogeneity led to a challenging data synthesis and study comparison, and restricted us to a qualitative (narrative) synthesis, rather than a quantitative synthesis in the form of a meta-analysis.

To enable a meaningful comparison of the studies’ results, we needed to introduce a rigorous approach to summarising them. First, we developed a working definition of the concepts explored in our research question. Despite widely acknowledged psychosocial stress models [4, 46], definitions of psychosocial stress and job stressors are not used consistently in the literature. This diversity in terminology of ‘stressor’ and ‘stress’ was apparent during several steps, such as during the construction of the search strategies and during data extraction. By framing these phenomena—for the purpose of this systematic review—as ‘work environment risk factors’ and ‘self-perceived stress outcomes’, we attempted to harmonize these differences in terminology. To be able to study relationships between stressors and stress, we classified measured variables into one of these two categories according to our working definitions (see Background). This allowed us to compare different studies’ results.

As mentioned, we disregarded the original studies’ framing of independent and dependent variables and instead classified them according to our own criteria. While this enabled us to look at the studies from the point of view of our study question, it introduced the risk of misrepresenting the original findings.

This concern was alleviated by limiting our data extraction of study results to correlation coefficients, which helped us increase study comparability at the same time. The advantage of considering only correlational analyses is that the Pearson correlation coefficient is a symmetric statistic. This allowed us to sidestep the original authors’ hypotheses and models and frame their (partial) results in our work environment risk factors and stress outcomes research question. This had the effect of including studies from different fields and getting an overview of these relationships, which was as broad as possible.

On the other hand, considering only correlational analysis led to an incomplete representation of results of several included studies. This is especially true of the studies performing more extensive analyses, since correlational analysis was merely an intermediate step before final conclusions based on multilevel analysis or analyses focusing on moderating or mediating effects. This has already been illustrated with the case of physiological responses. With such a diverse set of predictors and outcomes, however, comparing the results of these more complex models proved to be problematic, since it is impossible to compare specific effects without considering the full model.

Another consideration is the focus on statistical significance of correlations. While a fixation on statistical significance has been widely criticised [47], it served as a good first step in comparisons of heterogeneous studies. Effect size examination made little sense as all the reported within-subject correlation coefficients were low (i.e., *r*<0.5). Noting the above points about simplification with regard to results reporting, the raw number of statistically significant and nonsignificant correlations should still serve as a first overview of the field.

## Conclusions

While the field of chronic stress in the workplace is very well established, how daily work situations translate to day-to-day experience of stress and later to chronic conditions seems to be less understood.

We identified several high-quality studies dealing with this topic. The models they employ and the analytical methods they use are well developed. However, their research questions are particular and usually involve a somewhat narrow definition of stress. Instead of approaching stress outcomes as manifestations of a multifaceted response, only some types of responses are considered, most often affective responses. In our review, none of the studies approached this topic from a full-fledged stress model that would incorporate all the relevant aspects of the response to stressors.

Such a study would first require a combination of various data collection methods, such as ecological momentary assessment and continuous physiological monitoring. It would also call for a more complex analysis approach, such as combining multilevel modelling with structural equation modelling or other probabilistic graphical models. Finally, it would need to deal with the problem in the context of a well-established model of stress that lends itself well to such modelling.

## Supplementary Information

**Additional file 1** Tables 1 and 2 show work environment risk factors and stress outcomes, respectively, together with the tools used to measure them in original studies. They are ordered in broader categories according to the 6th European Working Conditions Survey [29] and with respect to the stress model of Ice and James [30].

**Additional file 2** Correlations were extracted from studies that reported them. They are listed in a spreadsheet to enable replication of analyses. These correlations were counted to produce Fig. 2 in the main body of text.

**Additional file 3** The full search strategies with indexing terms and free text words for all the databases searched: PubMed, Embase, Web of Science, Scopus, CINAHL, ERIC, and PsycArticles.

**Additional file 4** The PRISMA Checklist noting the page numbers of all necessary review items.

## Data Availability

All data generated or analysed during this study are included in this published article and its Additional Files. Additional evaluations done by two reviewers independently are available from the corresponding author on reasonable request.

## Abbreviations

EMA: Ecological momentary assessment
PANAS: Positive affect negative affect scale
