# Supplementary material for: Work environment risk factors causing day-to-day stress in occupational settings: a systematic review
Source: BMC Public Health. 2022 Feb 5;22:240. doi: 10.1186/s12889-021-12354-8 (PMC8818147; doi:10.1186/s12889-021-12354-8)
Supplement: Supplementary file 1 — Additional file 1 Tables 1 and 2 show work environment risk factors and stress outcomes, respectively, together with the tools used to measure them in original studies. They are ordered in broader categories according to the 6th European Working Conditions Survey [29] and with respect to the stress model of Ice and James [30]. [file 12889_2021_12354_MOESM1_ESM.pdf]

1 Categorization of work environment factors and stress outcomes

1.1 Work environment risk factors

Table 1: Work environment risk factors and their measurement tools categorized according to the 6th European Working Conditions Suvey [29].

ID	Study reference	Description and measurement tool(s)
		**Work intensity**
3	[50]	Perceived workload/work demand: Physical demand, mental demand, frustration effort, perceived time pressure, level of performance, measured with National Aeronautics and Space Administration-Task Load Index (NASA-TLX), 10-point Likert scale ranging from ‘low’ (0) to ‘high’ (9).
5	[52]	Workload: Perceived Workload Scale, 3 items.
12	[58]	Daily role conflict: 1 item from an adapted version of the Role Conflict and Ambiguity Scale, scale ranging from ‘I strongly disagree’ (1) to ‘I strongly agree’ (5).
14	[60]	Task demand: Diary of Ambulatory Behavioral States (DABS), 4-point scale ranging from ‘no!!’ to ‘yes!!’.
15	[61]	Job demands: Emotional demands: Ranging from ‘never’ (0) to ‘almost always’ (6). Over the past 3 days. Workload: 2 items of the Job Content Questionnaire (JCQ), 7-point Likert scale ranging from ‘strongly disagree’ (1) to ‘strongly agree’ (7). Over the past 3 days.
16	[62]	Daily workload: 3 items, 7-point scale ranging from ‘totally disagree’ (1) to ‘totally agree’ (7).
18	[64]	Demand: 1 question, how far it can be applied to the characteristics of the task performed at the time of measurement. Wide screen visual analogue scale ranging from ‘0’ to ‘10’ (to evaluate intensity).
18	[64]	Effort: 1 question, how far it can be applied to the characteristics of the task performed at the moment of measurement. Wide screen visual analogue scale ranging from ‘0’ to ‘10’ (to evaluate intensity).
18	[64]	Perceived workload/work demand: Mental demands, physical demands, time pressure, frustration, effort, performance, measured with NASA-TLX, 10-point Likert scale ranging from ‘low’ (0) to ‘high’ (9).
18	[64]	Time pressure: 4 items of the Instrument for Stress Oriented Task Analysis, 5-point scale ranging from ‘very seldom/never’ (1) to ‘very often’ (5).

Table 1: Work environment risk factors and their measurement tools categorized according to the 6th European Working Conditions Suvey [29].

ID	Study reference	Description and measurement tool(s)
20	[66]	Time pressure: 4 items of the Instrument for Stress Oriented Task Analysis, 5-point scale ranging from 'very seldom/never' (1) to 'very often' (5).
22	[68]	Workflow interruptions: Estimation of the number of interruptions during the previous 30 minutes, by sources (doctors, nurses, patients, assistants, technical problems, telephone).
22	[68]	Mental demands: 1) measured during the shift, 1 item from the NASA-TLX, regarding the previous 30 minutes, 20-point scroll bar ranging from 'very low' (1) to 'very high' (20). 2) measured in the evening, 3 items from the Instrument for Stress-related Task Analysis (ISTA) about concentration demands, 5-point Likert scale ranging from 'strongly disagree' (1) to 'strongly agree' (5).
22	[68]	Time pressure: 1) measured during the shift, 1 item from the NASA-TLX, regarding the previous 30 minutes, 20-point scroll bar ranging from 'very low' (1) to 'very high' (20). 2) measured in the evening, 3 items from the ISTA about time pressure, 5-point Likert scale ranging from 'strongly disagree' (1) to 'strongly agree' (5).
26	[72]	Demands: 9-point Likert scale, ranging from 'low' (1) to 'high' (9).
28	[74]	Perceived workload/work demand: Mental demands, physical demands, time pressure, frustration, effort, performance, measured with NASA-TLX, 10-point Likert scale ranging from 'low' (0) to 'high' (9).
29	[75]	Time pressure: 3 items of the Instrument for Stress-Oriented Analysis of Work Scale, 5-point Likert scale ranging from 'strongly disagree' (1) to 'strongly agree' (5).
29	[75]	Working faster and working longer: 2 items each, Self-Endangering Work Behavior Scale, either 'no' (0) or 'yes' (1).
32	[78]	Level of subjective pressure: 7-point scale ranging from 'extreme pressure' (1) to 'very little pressure' (7).
33	[79]	Demand: 5 items, scores ranging from '0' to '1'.
33	[79]	Effort: 4 items, scores ranging from '0' to '1'.
35	[80]	Job demands: JCQ, 4 items, ranging from 'strongly disagree' (1) to 'strongly agree' (5).

Table 1: Work environment risk factors and their measurement tools categorized according to the 6th European Working Conditions Suvey [29].

ID	Study reference	Description and measurement tool(s)
37	[82]	Workload/work demands: 4 items, conflicting demands on their time, varying levels of demand on their time, difficult work tasks, work tasks that they would prefer not to do. 5-point response scale ranging from 'not at all' (1) to 'a great deal' (5).
38	[83]	Workload: Mental demands, physical demands, temporal demands, operator performance, effort, frustration, measured with NASA-TLX.
39	[84]	Job demands: 1 item, Likert-type scale, ranging from 'not demanding at all' (0) to 'extremely demanding' (10).
		**Working time quality**
2	[49]	Time spent daily on work activities: List of 12 work activities, time spent on it (0: none, 1: <1 hour, 2: 1 to 2 hours, ... 7: >6 hours).
2	[49]	Overtime work: Time spent on all 12 work activities after 18.00 during weekdays and before and after 18:00 during weekends.
5	[52]	Day-to-day cross-domain ICT usage at the workplace: 4 items, 5-point scale ranging from 0 min to 5 min (1), 6 min to 15 min, 16 min to 30 min, 31 min to 60 min, to 60 min or more (5).
5	[52]	Segmentation preference: Segmentation Preference Scale, 4 items.
6	[53]	Family interference with work in the morning: 6 items, occurrence of the events rated either 'yes' (1) or 'no' (0).
34	[37]	Work-to-family conflict: Work-To-Family Conflict Scale, 5 questions, responses ranging from 'not at all' (1) to 'a lot' (4).
35	[80]	Daily working hours: In hours and minutes, 1 item.
37	[82]	Hours worked: Total number of hours worked per week.
37	[82]	Work-nonwork interference: Work to nonwork interference and nonwork to work interference. 5-point response scale ranging from 'not at all' (1) to 'a great deal' (5).
41	[86]	Work hours: In the past 24 hours.

Table 1: Work environment risk factors and their measurement tools categorized according to the 6th European Working Conditions Suvey [29].

ID	Study reference	Description and measurement tool(s)
		**Social environment**
4	[51]	Interpersonal conflict at work: 5-item checklist describing events: 'I had a fight with a co-worker over a work-related issue'. 'Co-worker(s) showed disapproval of the way I handled a work situation'. 'A colleague took jabs at or needled me'. 'Had to explain an improper behavior or action to co-worker(s) and/or supervisor'. 'Supervisor showed disapproval of the way I handled a work situation'. During the last 3 hours.
4	[51]	Social support: 6-item checklist: 'Co-worker helped with a certain task or problem'. 'Co-worker or customer gave information that helped me in my work'. 'Co-worker gave advice on how to handle things at work'. 'Co-worker gave his/her opinion on a problem concerning my work'. 'Co-worker explained how to perform a certain task or activity'. 'Supervisor gave advice on how to deal with a certain co-worker or customer'. During the last 3 hours.
7	[54, 76, sub-study 1]	Social interaction record: 13 rating scales about emotional concern, instrumental aid, information, appraisal, companionship. 5-point scale ranging from 'not' (1) to 'to a large extent' (5). Participants described the most significant interactions (maximum 5) with a minimum duration of 10 minutes (moment when the contact started, duration of contact, whether the other was a colleague, superior, subordinate, rating of the content: rewarding interactions, such as companionship and intimacy, 4 elements of support: emotional, appraisal, information, instrumental).
8	[36]	Co-worker support: 1 item, 5-point Likert scale ranging from 'not at all' (1) to 'very much' (5).
9	[55]	Daily customer-related social stressors: 16-item scale measuring 4 stressor groups (disproportionate customer expectations, customer verbal aggressions, ambiguous customer expectations, disliked customers), 5-point agreement scale ranging from 'not at all true' (1) to 'totally true' (5).
11	[57]	Daily incivility: Uncivil behaviour from supervisors, co-workers and customers, 20-item checklist (10 items from the Interpersonal Treatment at Work Scale and 10 items from the Uncivil Workplace Behavior Questionnaire). Reported if it was experienced on that day, either 'yes' or 'no'.

Table 1: Work environment risk factors and their measurement tools categorized according to the 6th European Working Conditions Suvey [29].

ID	Study reference	Description and measurement tool(s)
12	[58]	Daily transformational leadership: 6 items of the Transformational Leadership Inventory, includes 6 facets: Identifying and articulating a vision, providing an appropriate model, fostering the acceptance of group goals, high performance expectations, providing individualized support, intellectual stimulation. Scale ranging from 'I strongly disagree' (1) to 'I strongly agree' (5).
12	[58]	Daily team cooperation: 1 item, scale ranging from 'I strongly disagree' (1) to 'I strongly agree' (5).
12	[58]	Type of communication with supervisor: 2 items, if direct and/or indirect communication occurred on that day.
13	[59]	Daily non-sanctioned political influence tactics: Intimidation, manipulation, blaming or attacking others, Non-Sanctioned Political Tactics Measure, 3 items, either 'no' (0) or 'yes' (1). And the daily frequency of the 3 categories.
14	[60]	Social interaction: Current or recent (within the last 10 minutes), 27 items from the DABS during every assessment. Either 'yes' or 'no'. If a social interaction was acknowledged, additional 31 items from the DABS were administered (including tone and content of the interaction).
14	[60]	Social conflict: Presence and intensity, DABS, 4-point scale ranging from 'no!!' to 'yes!!'. 'No!!' if no social interaction was reported.
15	[61]	Supervisor support: 2 items, 7-point Likert scale ranging from 'strongly disagree' (1) to 'strongly agree' (7).
15	[61]	Co-worker support: 2 items, 7-point Likert scale ranging from 'strongly disagree' (1) to 'strongly agree' (7).
15	[61]	Organisational support: 4 items, perceived Organisational Support Scale, 7-point Likert scale ranging from 'strongly disagree' (1) to 'strongly agree' (7).
18	[64]	Social stressors at work: Negative interactions with colleagues and supervisors, 10 items, 5-point Likert scale ranging from 'strongly disagree' (1) to 'strongly agree' (5).

Table 1: Work environment risk factors and their measurement tools categorized according to the 6th European Working Conditions Suvey [29].

ID	Study reference	Description and measurement tool(s)
19	[65]	Social interactions at work: Rochester Interaction Record, number of social contacts during the interval (of personal importance or a duration of more than 10 minutes), face-to-face or electronically, interaction partner (co-worker, collaborator, supervisor, client) and rating of quality via 7-point response scale ranging from ‘very agreeable’ to ‘very disagreeable’ and from ‘very unburdening’ to ‘very burdening’ (severity of negative social interactions at work).
20	[66]	Social stressors at work: Negative interactions with colleagues and supervisors, 10 items, 5-point Likert scale ranging from ‘strongly disagree’ (1) to ‘strongly agree’ (5).
23	[69]	Verbal interaction: Average speaking length and total duration of voice activity (speaking segments).
26	[72]	Social support: 9-point Likert scale, ranging from ‘low’ (1) to ‘high’ (9).
30	[76, sub-study 2]	Social contact record: Participants described the most significant interactions (maximum 5) with a minimum duration of 10 minutes (duration of contact, with whom it occurred, rating of the content: rewarding interactions, such as companionship and intimacy, 4 elements of support: emotional, appraisal, information, instrumental, indication whether the relationship was perceived as reciprocal in regards of supporting each other).
30	[76, sub-study 3]	Social contact record: Participants described the most significant interactions (maximum 5) with a minimum duration of 10 minutes (duration of contact, with whom it occurred, rating of the content: rewarding interactions, such as companionship and intimacy, 4 elements of support: emotional, appraisal, information, instrumental, indication whether feelings of inferiority as a consequence of supporting each other were experienced: threat to self-esteem).
34	[37]	Supervisor support: National Study of Daily Experiences, 2 items, scale ranging from 1 to 7.
36	[81]	Daily laissez-faire leadership: 2 items, 5-point scale, ranging from ‘strongly disagree’ (1) to ‘strongly agree’ (5).
37	[82]	Social support: Support of clients, family and friends, and colleagues and peers, 3-item formative scale.

Table 1: Work environment risk factors and their measurement tools categorized according to the 6th European Working Conditions Suvey [29].

ID	Study reference	Description and measurement tool(s)
		**Skills and discretion**
6	[53]	Daily task significance: 4 items, measured with a 7-point Likert scale ranging from 'strongly disagree' (1) to 'strongly agree' (7).
14	[60]	Decisional control: DABS, 4-point scale ranging from 'no!!' to 'yes!!'.
15	[61]	Autonomy: 2 items of the Self-Determination Scale, 7-point Likert scale ranging from 'strongly disagree' (1) to 'strongly agree' (7).
18	[64]	Control: (autonomy and skills development), 1 question, how far it can be applied to the characteristics of the task performed at the moment of measurement. Wide screen visual analogue scale ranging from '0' to '10' (to evaluate intensity).
20	[66]	Daily illegitimate tasks: 8 items of the Bern Illegitimate Tasks Scale, 5-point Likert scale ranging from 'very rarely/never' (1) to 'very often' (5).
26	[72]	Control: 9-point Likert scale, ranging from 'low' (1) to 'high' (9).
33	[79]	Control: 3 items, scores ranging from '0' to '1'.
37	[82]	Work control: Timing control and method control, 7-item reflective scale.
39	[84]	Self-concordant motivation: Identified and integrated motivation. Likert-type scale, ranging from 'not at all for this reason' (0) to 'completely for this reason' (10).
40	[85]	Situational control: 1 item, ranging from 'very small' (1) to 'very large' (6).
		**Prospects**
18	[64]	Reward: 1 question, how far it can be applied to the characteristics of the task performed at the moment of measurement. Wide screen visual analogue scale ranging from '0' to '10' (to evaluate intensity).
24	[70]	General workplace uncertainty: 4-item scale ranging from 'strongly disagree' (1) to 'strongly agree' (5).
33	[79]	Reward: 3 items, scores ranging from '0' to '1'.

Table 1: Work environment risk factors and their measurement tools categorized according to the 6th European Working Conditions Suvey [29].

ID	Study reference	Description and measurement tool(s)
		**Commuting from and to the workplace**
6	[53]	Morning commuting stressors: Checklist (created through a focus group) with 25 possible stressful events. Either ‘this event happened this morning’ or ‘this event did not happen this morning’.
35	[80]	Stressful delays during commuting: If they experienced any form of delay or traffic congestion during the commute home, either ‘no’ (0) or ‘yes’ (1), if ‘yes’, they rated the stressfulness with 3 items, 5-point scale ranging from ‘totally disagree’ (1) to ‘totally agree’ (5).
35	[80]	Commuting time: Time (in minutes) they spent commuting by car, public transport, bike or walk, other means.
35	[80]	Recovery experiences during the commuting: Recovery Experience Questionnaire (detachment, relaxation, mastery), 4 items each, 5-point Likert scale ‘strongly disagree’ (1) to ‘strongly agree’ (5).
		**Occupation-specific (medicine and health care)**
17	[63]	Ambulance alarms: Recorded in journals.
21	[67]	Clinical workload: Patient load, admission volume, familiarity with patients, type of call day, continuity clinic attendance, etc., reported by a series of survey questions.
27	[73]	Number of emergency calls: Number of calls occurring during the previous 24 hours. Emergency Call Questionnaire (ECQ), ECQ number (number of calls).
28	[74]	Clinical workload: Number of patients assigned, nurse’s rating of familiarity with the patient on a scale ranging from ‘0’ to ‘9’ (higher rating representing greater familiarity), facility (adult or paediatric), setting (intensive care unit or acute care), work schedule (average length of workday), clinical experience (number of months on the unit), work activities/type of work (direct care, indirect care, administrative, education, personal, other).
38	[83]	Clinical workload: System workload metrics (via emergency department information system), patient quantity and patient complexity (chief complaint, patient wait time, patient length of stay, patient acuity at triage, managing physician, number of patients in the waiting room, ED occupancy, diversion status, average wait times, length of stay for all patients).

Table 1: Work environment risk factors and their measurement tools categorized according to the 6th European Working Conditions Suvey [29].

ID	Study reference	Description and measurement tool(s)
38	[83]	Clinical workload: Observational task analysis (by trained observer), recording of type and duration of predefined primary clinical tasks, work interruptions.
		**Various**
1	[48]	Daily hassles: Hassles occurring during a shift and hassles affecting participants on a shift. Open-response format. Time was recorded. Perceived intensity of daily hassles was measured with a scale ranging from ‘not stressful’ (0) to ‘very stressful’ (4).
7	[54, 76, sub-study 1]	Stressful events record: Participants described the 5 most stressful events (events which made them feel upset for 2 hours or more). Examples of police work were given.
10	[56]	Stressful situations: The most stressful situation during the working day. Open-response format.
17	[63]	Cause of current heart rate: Information of possible causes of current heart rate. Open-response format.
18	[64]	Nursing task: Work Observation Method by Activity Timing (WOMBAT) classification: direct care, medication tasks, documentation, other professional tasks, resting.
21	[67]	Description of work activities: Classified as direct patient care, indirect care, education, transit, personal. Measured via EMA.
25	[71]	Stressful situations: COMES stress monitoring system, verbal description of the episode and situational appraisals, and emotional stress reactions.
26	[72]	Daily hassles and uplifts: 24 items, 6 items per category hassles/uplifts/work/non-work incl. commuting. 3-point scale, ranging from ‘not at all’ (0), to ‘a little’ (1), to ‘a lot’ (2).
26	[72]	Menstrual symptoms: 2 symptoms, 3-point scale, ranging from ‘not at all’ (0), to ‘a little’ (1), to ‘a lot’ (2).
27	[73]	Number of stressful events: Minor stressors, whether the event occurred during the previous 24 hours. Daily Stress Inventory (DSI), DSI event (number of stressful events).
30	[76, sub-study 2]	Stressful events record: Participants described the 5 most stressful events (events which made them feel upset for 2 hours or more).

Table 1: Work environment risk factors and their measurement tools categorized according to the 6th European Working Conditions Suvey [29].

ID	Study reference	Description and measurement tool(s)
30	[76, sub-study 3]	Stressful events record: Participants described the 5 most stressful events (events which made them feel upset for 2 hours or more).
31	[77]	Type and time of daily activities: Noted in a personal logbook.
33	[79]	Nursing work tasks: WOMBAT, 10 categories: direct patient care, indirect care, medication, documentation, professional communication, in transit, social/break, supervision, ward related and other (tasks within the preceding 10 minutes).
34	[37]	Work-related stressors: Incidence of any kind of work-related stressor on a given day (work demands, argument or disagreement, any other stressful events happening at work), either ‘no’ (0) or ‘yes’ (1).
39	[84]	Daily work activities: The two activities with which they spent the most time during the working day. Choosing from 11 work activities in a drop-down list and/or open-response format. And duration spent on these activities was noted.
40	[85]	Stressful situations/events: Content of every stressful experience (minor and major). Open-response format and items concerning the characteristics of the situation/event (similarity with previous experiences, probability of reoccurrence).
41	[86]	Daily stressors: Daily Inventory of Stressful Events, work-related stressors in the past 24 hours, work arguments, interpersonal tensions, employee or co-worker related stressors, stressors involving hotel guests, general work overloads (content, who was involved, perceived threat, severity, appraisal), either ‘no stressor that day’ (0) or ‘stressor’ (1).

1.2 Stress outcomes

Table 2: Stress outcomes and their measurement tools categorized according to the adapted stress model of Ice and James [30].

ID	Study reference	Description and measurement tool(s)
		**Affective response**
2	[49]	Work experiences: Pleasurable, effortful, and stressful. Ranging from 'not at all' (1) to 'extremely' (10).
3	[50]	Emotional stress: Feelings of tension, alertness, anger, sadness, fatigue, stress, unhappiness, tired, worried, upset, based on 3 items of the Diary of Ambulatory Behavioral States (DABS), 10-point Likert scale ranging from 'low' (0) to 'high' (9). Time stamp (date, hour, minute) was recorded.
4	[51]	Negative affect: 10 adjectives of the Positive and Negative Affect Scale (PANAS), at the time of survey completion.
5	[52]	Daily stress: 3 items of the Oldenburg Burnout Inventory, measures feeling of emptiness, overwork, and physical exhaustion.
7	[54, 76, sub-study 1]	Daily negative affect: 11-item scale measuring the frequency of emotions (irritated, depressed, confused, nervous), 5-point scale ranging from 'not' (1) to 'to a very large extent' (5).
9	[55]	Negative affect: 6 items (distressed, upset, irritable, nervous, jittery, afraid) of the PANAS, 5-point agreement scale ranging from 'not at all' (1) to 'totally' (5).
11	[57]	Daily stress: 7-item stress subscale (of the short version from the Depression Anxiety Stress Scale), measures distress, tension, irritability, self-reported tendency to overreact to stressful events.
12	[58]	Daily stress: 3 items from the Irritation Scale, scale ranging from 'I strongly disagree' (1) to 'I strongly agree' (5).
13	[59]	Daily negative affect activation: Negative Affect Scale (shortened version), scale ranging from 'not at all' (1) to 'very much' (5).
14	[60]	Emotional activation: Negative affect and arousal, DABS, 4-point scale ranging from 'no!!' to 'yes!!'.
15	[61]	Emotional exhaustion: Ranging from 'never' (0) to 'almost always' (6). Over the past 3 days.
18	[64]	Hedonic tone: 1-item visual analogue scale of 5 points ranging from 'happy face' to 'sad face'.

Table 2: Stress outcomes and their measurement tools categorized according to the adapted stress model of Ice and James [30].

ID	Study reference	Description and measurement tool(s)
18	[64]	Fatigue: 1-item visual analogue scale of 5 points ranging from ‘full battery’ to ‘empty battery’.
19	[65]	High-arousal negative affect: Mohr Irritation Scale, measures ‘angry’ and ‘irritated’.
19	[65]	Low-arousal negative affect: Multi-dimensional Affect Questionnaire, measures e.g., ‘bad’ and fatigue dimension, measures e.g., ‘weary’.
20	[66]	State negative affect: 12 items of the PANAS Expanded Form (PANAS-X) scale, 5-point scale ranging from ‘strongly disagree’ (1) to ‘strongly agree’ (5).
21	[67]	Emotional stress: 10-questions (unhappy, tired, tense, alert, worried, stressed, etc.) from the DABS, 10-point Likert-like scale ranging from ‘0’ to ‘9’ (higher numbers indicating worse stress). (Measured via EMA)
22	[68]	Satisfaction of the employee with his/her performance: Regarding work, 1 item, 5-point Likert scale ranging from ‘strongly disagree’ (1) to ‘strongly agree’ (5).
22	[68]	Emotional Irritation: Regarding work, 7-point Likert scale ranging from ‘strongly disagree’ (1) to ‘strongly agree’ (7).
23	[69]	Stress level: 1 question (‘What is your stress level?’).
23	[69]	Negative affect: Profile of Mood States Scale (POMS), angry, tense, anxious, sad. 5-point scale ranging from ‘very slightly or not at all’ (1) to ‘extremely’ (5).
23	[69]	Positive affect: POMS, friendly, effective, energetic, cheerful. 5-point scale ranging from ‘very slightly or not at all’ (1) to ‘extremely’ (5).
24	[70]	Stress: 4-item scale ranging from ‘strongly disagree’ (1) to ‘strongly agree’ (5).
24	[70]	Job dissatisfaction: 5 items of the Job Satisfaction Scale (reverse-coded), ranging from ‘strongly disagree’ (1) to ‘strongly agree’ (5).
24	[70]	Emotional exhaustion: 5-item scale ranging from ‘never’ (1) to ‘very frequently’ (5).
24	[70]	Daily negative affect: 5 items of the PANAS.
25	[71]	Momentary psychological state or functioning: 11-point scale ranging from ‘very … (negative pole)’ (-5) to ‘very … (positive pole)’ ($+5$), mood, mental functioning, physical energy, tension, and perceived level of stress.

Table 2: Stress outcomes and their measurement tools categorized according to the adapted stress model of Ice and James [30].

ID	Study reference	Description and measurement tool(s)
26	[72]	Subjective strain: 16 items, 9-point Likert scale, ranging from 'did not feel that way at all' (1) to 'felt that way very much (9)'.
27	[73]	Perceived stressfulness: Rating each event, 7-point Likert-type scale ranging from 'occurred but was not stressful' (1) to 'caused me to panic' (7). Daily Stress Inventory (DSI), DSI impact (sum of perceived stressfulness).
27	[73]	Stressfulness: Rating each call, 7-point Likert-type scale ranging from 'occurred but was not stressful' (1) to 'caused me to panic' (7). Emergency Call Questionnaire (ECQ), ECQ impact (sum of stressfulness).
28	[74]	Stress: 10-item questionnaire, different emotional activation states, anger, worry, tension, alert, fatigue, unhappy, tired, upset, sad, stress, 10-point Likert scale ranging from 'low' (0) to 'high' (9).
29	[75]	Emotional irritation: During the day, 8 items, 7-point Likert scale ranging from 'strongly disagree' (1) to 'strongly agree' (7).
30	[76, sub-study 2]	Daily negative affect: Questionnaire assessing the presence of negative feelings.
30	[76, sub-study 3]	Daily negative affect: Questionnaire assessing the presence of negative feelings.
31	[77]	Stress: Short version of State Trait Anxiety Inventory (STAI), measures emotional, cognitive, physical stress, 6 items, 4-point scale ranging from 'not at all' to 'very much'.
33	[79]	Mood/experienced stress: University of Wales Institute of Science and Technology Mood Scale (UWIST), experienced stress, affect, fatigue. Visual analogue scales ranging from 'no' (0) to 'yes' (100).
34	[37]	Negative affect: 10 items (scared, afraid, upset, distressed, jittery, nervous, ashamed, guilty, irritable, hostile) for negative affect of the PANAS, responses ranging from 'none of the time' (1) to 'all of the time' (5).
35	[80]	Anxiety: PANAS-X, 6 items, 5-point Likert scale 'not at all' (1) to 'extremely' (5).
35	[80]	Serenity: Serenity subscale of the PANAS-X, 5-point Likert scale ranging from 'not at all' (1) to 'extremely' (5).
36	[81]	Daily stress: 2 items, 5-point scale, ranging from 'strongly disagree' (1) to 'strongly agree' (5).

Table 2: Stress outcomes and their measurement tools categorized according to the adapted stress model of Ice and James [30].

ID	Study reference	Description and measurement tool(s)
37	[82]	Well-being: 2 items about calmness (anxious vs. calm) and 2 items about enthusiasm (gloomy/depressed vs. enthusiastic), 5-point response scale ranging from ‘not at all’ (1) to ‘a great deal’ (5). Responses for anxious and gloomy/depressed were reverse-coded.
39	[84]	Happiness: 1 item, scale ranging from ‘not happy at all’ (0) to ‘very happy’ (10).
40	[85]	Stress intensity: Measured on a 6-point Likert scale ranging from ‘very low’ (1) to ‘very high’ (6).
41	[86]	Negative affect: PANAS measuring distressed, upset, guilty, scared, hostile, irritable, ashamed, nervous, jittery, afraid. 5-point scale ranging from ‘very slightly/not at all’ (0) to ‘extremely’ (4).
		**Appraisal**
1	[48]	Perceptions of patient safety: Measured in relation to ‘this shift’ with the Hospital Survey on Patient Safety Culture (HSOPC). 4 items, 5-point scale ranging from ‘strongly disagree’ (1) to ‘strongly agree’ (5).
1	[48]	Safe practitioner measure: Measured in relation to ‘this shift’. 1 item, 5-point scale ranging from ‘strongly disagree’ (1) to ‘strongly agree’ (5).
6	[53]	Morning commuting strain: 8 adjectives from the Stress in General Scale to describe their feelings during commuting with a 5-point Likert scale ranging from ‘strongly disagree’ (1) to ‘strongly agree’ (5).
10	[56]	Stress intensity: Indicating how much stress was experienced during the stressful situation, 5-point Likert-type scale ranging from ‘low’ (1) to ‘high’ (5).
13	[59]	Daily threat appraisal: Threat, hindrance, obstruction, 3 items, 5-point scale ranging from ‘strongly disagree’ (1) to ‘strongly agree’ (5).
		**Behavioural response**
5	[52]	Daily job performance: 3 items of the Tarafdar, Tu, Ragu-Nathan, and Ragu-Nathan Productivity Questionnaire.
6	[53]	Self-regulation during morning work: 12 items, measured with a 5-point Likert scale.

Table 2: Stress outcomes and their measurement tools categorized according to the adapted stress model of Ice and James [30].

ID	Study reference	Description and measurement tool(s)
24	[70]	Counterproductive work behavior: 6 items of the Counterproductive Work Behavior toward the Supervisor Scale, ranging from 'strongly disagree' (1) to 'strongly agree' (5).
26	[72]	Job performance: 3 items, 9-point Likert scale, ranging from 'not very' (1) to 'very' (9).
		**Cognitive outcome**
1	[48]	Workplace cognitive failure: Measured in relation to 'this shift' with the Workplace Cognitive Failure Scale (WCFS). 15 items, 5-point scale ranging from 'never' (0) to 'very often' (4).
20	[66]	Lack of psychological detachment from work: 4-item scale, 5-point scale ranging from 'strongly disagree' (1) to 'strongly agree' (5). Measured shortly before going to bed.
22	[68]	Forgetting of intentions: Regarding work, 1 item, either 'no' (0) or 'yes' (1).
22	[68]	Cognitive Irritation: Regarding work, 7-point Likert scale ranging from 'strongly disagree' (1) to 'strongly agree' (7).
29	[75]	Cognitive irritation: During the day, 8 items, 7-point Likert scale ranging from 'strongly disagree' (1) to 'strongly agree' (7).
		**Health outcome**
26	[72]	Psychosomatic symptoms: 15 items, 3-point scale, ranging from 'not at all' (0), to 'a little' (1), to 'a lot' (2).
41	[86]	Health symptoms: Symptom checklist measuring aches, gastrointestinal symptoms, upper respiratory symptoms. Indicating if they had each symptom, either 'no' (0) or 'yes' (1) and their severity ranging from 'very mild' (1) to 'very severe' (10).
		**Motivational response**
11	[57]	Work engagement: 9 items (shortened Utrecht Work Engagement Scale), includes 3 aspects: Vigour, dedication, absorption. 5-point Likert-type scale ranging from 'strongly disagree' (1) to 'strongly agree' (5).
15	[61]	Work engagement: Ranging from 'never' (0) to 'almost always' (6). Over the past 3 days.
16	[62]	Work engagement: 9 items of the Utrecht Work Engagement Scale, 7-point scale ranging from 'totally disagree' (1) to 'totally agree' (7).

Table 2: Stress outcomes and their measurement tools categorized according to the adapted stress model of Ice and James [30].

ID	Study reference	Description and measurement tool(s)
29	[75]	Work engagement: 9 items of the Utrecht Work Engagement Scale, 7-point Likert scale ranging from ‘totally disagree’ (1) to ‘strongly agree’ (5).
		**Physiological response**
8	[36]	Heart rate variability: Continuously from Monday morning until Saturday morning (whole study period).
14	[60]	Systolic blood pressure: Every 45 minutes, during waking hours.
14	[60]	Diastolic blood pressure: Every 45 minutes, during waking hours.
14	[60]	Heart rate: Every 45 minutes, during waking hours.
17	[63]	Heart rate: Every 15 seconds, at work and at home; 24 hours a day.
19	[65]	Salivary cortisol: Before and after work.
31	[77]	Heart rate variability: During all daily work activities.
31	[77]	Stress percentage: Displayed every 4 seconds, stress level ranging from ‘lowest’ (0) to ‘highest’ (100), no measurement during physical activity.
32	[78]	Systolic blood pressure: Every 20 minutes.
32	[78]	Diastolic blood pressure: Every 20 minutes.
32	[78]	Heart rate: Every 20 minutes.
33	[79]	Heart rate: Every 15 seconds, continuously over 2 shifts.
34	[37]	Salivary cortisol: 5 samples throughout the day.
38	[83]	Galvanic skin response: Measuring workload, minute-by-minute measurement, during the observation period.

References

[1] Fishta A, Backé EM. Psychosocial stress at work and cardiovascular diseases: An overview of systematic reviews. Int Arch Occup Environ Health. 2015 Feb;88(8):997–1014.

[2] Harvey SB, Modini M, Joyce S, Milligan-Saville JS, Tan L, Mykletun A, et al. Can work make you mentally ill? A systematic meta-review of work-related risk factors for common mental health problems. Occup Environ Med. 2017 Jan;74(4):301–310.

[3] Magnavita N, Chirico F. New and Emerging Risk Factors in Occupational Health. Applied Sciences. 2020 dec;10(24):8906.

[4] Johnson JV, Hall EM. Job strain, work place social support, and cardiovascular disease: a cross-sectional study of a random sample of the Swedish working population. Am J Public Health. 1988 Oct;78(10):1336–1342.

[5] Johnson JV, Hall EM, Theorell T. Combined effects of job strain and social isolation on cardiovascular disease morbidity and mortality in a random sample of the Swedish male working population. Scand J Work Environ Health. 1989 Aug;15(4):271–279.

[6] Karasek R, Theorell T. Healthy work: Stress, productivity and the reconstruction of working life. New York: Basic books; 1990.

[7] Siegrist J. Adverse health effects of high-effort/low-reward conditions. J Occup Health Psychol. 1996;1(1):27–41.

[8] Demerouti E, Bakker AB, Nachreiner F, Schaufeli WB. The job demands-resources model of burnout. Journal of Applied Psychology. 2001;86(3):499–512.

[9] Hauke A, Flintrop J, Brun E, Rugulies R. The impact of work-related psychosocial stressors on the onset of musculoskeletal disorders in specific body regions: A review and meta-analysis of 54 longitudinal studies. Work & Stress. 2011 jul;25(3):243–256.

[10] Siegrist J, Rödel A. Work stress and health risk behavior. Scand J Work Environ Health. 2006 Dec;32(6):473–481.

[11] Magnavita N, Stasio ED, Capitanelli I, Lops EA, Chirico F, Garbarino S. Sleep Problems and Workplace Violence: A Systematic Review and Meta-Analysis. Frontiers in Neuroscience. 2019 oct;13(997):1–18.

[12] Sato H, ichiro Kawahara J. Selective bias in retrospective self-reports of negative mood states. Anxiety Stress Coping. 2011;24(4):359–367.

[13] Bell C, Johnston D, Allan J, Johnston M, Pollard B. Repeated real time measures of work stress in nurses may not relate to questionnaire accounts. Psychol Health. 2013 Jul;28(sup1 (EHPS 2013 Abstracts)):65–66.

[14] Shiffman S, Stone AA. Introduction to the special section: Ecological momentary assessment in health psychology. Health Psychol. 1998 Jan;17(1):3–5.

[15] Smyth JM, Stone AA. Ecological Momentary Assessment Research in Behavioral medicine. J Happiness Stud. 2003;4(1):35–52.

[16] Hurrell JJ. Organizational Stress Intervention. In: Barling J, Kelloway EK, Frone MR, editors. Handbook of Work Stress. SAGE Publications, Inc.; 2005. p. 623–646.

[17] Higgins JPT, Thomas J, Chandler J, Cumpston M, Li T, Page MJ, et al., editors. Cochrane Handbook for Systematic Reviews of Interventions. John Wiley & Sons; 2020.

[18] Moher D, Liberati A, Tetzlaff J, Altman DG. Preferred Reporting Items for Systematic Reviews and Meta-Analyses: The PRISMA Statement. PLoS Medicine. 2009 Jul;6(7):e1000097.

[19] Bolliger L, Lukan J, Pauwels NS, Luštrek M, De Bacquer D, Clays E. Work environment risk factors causing day-to-day stress in occupational settings: A systematic review. PROSPERO 2018 CRD42018105355; 2018. Available from: https://www.crd.york.ac.uk/prospero/display_record.php?ID=CRD42018105355.

[20] Csikszentmihalyi M, Larson R, Prescott S. The ecology of adolescent activity and experience. J Youth Adolescence. 1977 Sep;6(3):281–294.

[21] Gibbons CJ. Turning the Page on Pen-and-Paper Questionnaires: Combining Ecological Momentary Assessment and Computer Adaptive Testing to Transform Psychological Assessment in the 21st Century. Front Psychol. 2017 Jan;7.

[22] McGowan J, Sampson M, Salzwedel DM, Cogo E, Foerster V, Lefebvre C. PRESS – Peer Review of Electronic Search Strategies: 2015 Guideline Explanation and Elaboration (PRESS E&E). Ottawa: CADTH; 2016.

[23] Ouzzani M, Hammady H, Fedorowicz Z, Elmagarmid A. Rayyan—a web and mobile app for systematic reviews. Systematic Reviews. 2016 Dec;5(1).

[24] Kmet L, Lee R, Cook L. Standard Quality Assessment Criteria for Evaluating Primary Research Papers from a Variety of Fields. Edmonton, Alta: Alberta Heritage Foundation for Medical Research; 2004.

[25] Schünemann H, Brożek J, Guyatt G, Oxman A. Handbook for grading the quality of evidence and the strength of recommendations using the GRADE approach; 2013. Retrieved from https://training.cochrane.org/resource/grade-handbook.

[26] Heeren JK. Stress, hardiness, and burnout among psychiatric nurses working in hospital settings [PhD thesis]. University of Virginia; 1991.

[27] Northrop LME. Stress, social support, and burnout in nursing home staff [PhD thesis]. West Virginia University; 1996.

[28] Johnstone M. Time and Tasks: Teacher Workload and Stress; 1993. ED368700. Spotlights 44.

[29] Eurofound. Sixth European Working Conditions Survey – Overview report (2017 update). Luxembourg: Publications Office of the European Union; 2017.

[30] Ice GH, James GD. Conducting a field study of stress. In: Ice GH, James GD, editors. Measuring Stress in Humans. Cambridge, UK: Cambridge University Press; 2006. p. 3–24.

[31] Watson D, Clark LA, Tellegen A. Development and validation of brief measures of positive and negative affect. J Pers Soc Psychol. 1988;54(6):1063–1070.

[32] Pejtersen JH, Burr H, Hannerz H, Fishta A, Eller NH. Update on Work-Related Psychosocial Factors and the Development of Ischemic Heart Disease. Cardiol Rev. 2015;23(2):94–98.

[33] Theorell T, Hammarström A, Aronsson G, Bendz LT, Grape T, Hogstedt C, et al. A systematic review including meta-analysis of work environment and depressive symptoms. BMC Public Health. 2015 Aug;15(738).

[34] Burr H, Formazin M, Pohrt A. Methodological and conceptual issues regarding occupational psychosocial coronary heart disease epidemiology. Scand J Work Environ Health. 2016 Mar;42(3):251–255.

[35] Alberdi A, Aztiria A, Basarab A. Towards an automatic early stress recognition system for office environments based on multimodal measurements. J Biomed Inform. 2016 Feb;59:49–75.

[36] Baethge A, Vahle-Hinz T, Rigotti T. Coworker support and its relationship to allostasis during a workday: A diary study on trajectories of heart rate variability during work. J Appl Psychol. 2020 May;105(5):506–526.

[37] Almeida DM, Davis KD, Lee S, Lawson KM, Walter KN, Moen P. Supervisor Support Buffers Daily Psychological and Physiological Reactivity to Work-to-Family Conflict. J Marriage Fam. 2016 Feb;78(1):165–179.

[38] Martikainen P, Bartley M, Lahelma E. Psychosocial determinants of health in social epidemiology. Int J Epidemiol. 2002 Dec;31(6):1091–1093.

[39] Rugulies R. What is a psychosocial work environment? Scand J Work Environ Health. 2019 Nov;45(1):1–6.

[40] Kim TJ, von dem Knesebeck O. Is an insecure job better for health than having no job at all? A systematic review of studies investigating the health-related risks of both job insecurity and unemployment. BMC Public Health. 2015 Sep;15(1).

[41] Steptoe A, Kivimäki M. Stress and cardiovascular disease. Nat Rev Cardiol. 2012 Apr;9(6):360–370.

[42] Steptoe A, Kivimäki M. Stress and Cardiovascular Disease: An Update on Current Knowledge. Annu Rev Public Health. 2013 Mar;34(1):337–354.

[43] Belkic K, Landsbergis PA, Schnall PL, Baker D. Is job strain a major source of cardiovascular disease risk? Scandinavian Journal of Work, Environment & Health. 2004 apr;30(2):85–128.

[44] Kivimäki M, Steptoe A. Effects of stress on the development and progression of cardiovascular disease. Nat Rev Cardiol. 2018 Dec;15(4):215–229.

[45] Kivimäki M, Virtanen M, Elovainio M, Kouvonen A, Väänänen A, Vahtera J. Work stress in the etiology of coronary heart disease—a meta-analysis. Scandinavian Journal of Work, Environment & Health. 2006 dec;32(6):431–442.

[46] Siegrist J, Li J. Associations of Extrinsic and Intrinsic Components of Work Stress with Health: A Systematic Review of Evidence on the Effort-Reward Imbalance Model. Int J Environ Res Public Health. 2016 Apr;13(4):432.

[47] Wasserstein RL, Lazar NA. The ASA Statement on p-Values: Context, Process, and Purpose. Amer Statist. 2016 Apr;70(2):129–133.

[48] Louch G, O'Hara J, Gardner P, O'Connor DB. A Daily Diary Approach to the Examination of Chronic Stress, Daily Hassles and Safety Perceptions in Hospital Nursing. Int J Behav Med. 2017 May;24(6):946–956.

[49] Beckers DGJ, van Hooff MLM, van der Linden D, Kompier MAJ, Taris TW, Geurts SAE. A diary study to open up the black box of overtime work among university faculty members. Scand J Work Environ Health. 2008 Jun;34(3):213–223.

[50] Rutledge T, Stucky E, Dollarhide A, Shively M, Jain S, Wolfson T, et al. A real-time assessment of work stress in physicians and nurses. Health Psychol. 2009 Mar;28(2):194–200.

[51] Ilies R, Johnson MD, Judge TA, Keeney J. A within-individual study of interpersonal conflict as a work stressor: Dispositional and situational moderators. J Organ Behav. 2011 Jan;32(1):44–64. First published: 22 December 2010.

[52] Yeh YJY, Ma TN, Pan SY, Chuang PJ, Jhuang YH. Assessing potential effects of daily cross-domain usage of information and communication technologies. J Soc Psychol. 2019 Oct;160(4):465–478.

[53] Zhou L, Wang M, Chang CH, Liu S, Zhan Y, Shi J. Commuting stress process and self-regulation at work: Moderating roles of daily task significance, family interference with work, and commuting means efficacy. Pers Psychol. 2017 Mar;70(4):891–922.

[54] Buunk BP, Verhoeven K. Companionship and Support at Work: A Microanalysis of the Stress-Reducing Features of Social Interaction. Basic Appl Soc Psych. 1991 Sep;12(3):243–258.

[55] Dudenhöffer S, Dormann C. Customer-related social stressors and service providers' affective reactions. J Organ Behav. 2012 Sep;34(4):520–539.

[56] Rodrigues S, Kaiseler M, Queirós C, Basto-Pereira M. Daily stress and coping among emergency response officers: a case study. Int J Emerg Serv. 2017 Aug;6(2):122–133.

[57] Beattie L, Griffin B. Day-level fluctuations in stress and engagement in response to workplace incivility: A diary study. Work Stress. 2014 Apr;p. 1–19.

[58] Diebig M, Bormann KC, Rowold J. Day-level transformational leadership and followers' daily level of stress: A moderated mediation model of team cooperation, role conflict, and type of communication. Eur J Work Organ Psy. 2017 Nov;26(2):234–249.

[59] Webster JR, Adams GA, Maranto CL, Beehr TA. "Dirty" Workplace Politics and Well-Being. Psychol Women Quart. 2018 May;42(3):361–377.

[60] Kamarck TW, Shiffman SM, Smithline L, Goodie JL, Paty JA, Gnys M, et al. Effects of task strain, social conflict, and emotional activation on ambulatory cardiovascular activity: Daily life consequences of recurring stress in a multiethnic adult sample. Health Psychol. 1998;17(1):17–29.

[61] Albrecht SL, Anglim J. Employee engagement and emotional exhaustion of fly-in-fly-out workers: A diary study. Aust J Psychol. 2018 Feb;70(1):66–75.

[62] Breevaart K, Bakker AB, Derks D, van Vuuren TCV. Engagement during demanding workdays: A diary study on energy gained from off-job activities. Int J Stress Manage. 2020 Feb;27(1):45–52.

[63] Karlsson K, Niemelä P, Jonsson A. Heart Rate as a Marker of Stress in Ambulance Personnel: A Pilot Study of the Body's Response to the Ambulance Alarm. Prehospital Disaster Med. 2011 Feb;26(1):21–26.

[64] Fernández-Castro J, Martínez-Zaragoza F, Rovira T, Edo S, Solanes-Puchol Á, del Río BM, et al. How does emotional exhaustion influence work stress? Relationships between stressor appraisals, hedonic tone, and fatigue in nurses' daily tasks: A longitudinal cohort study. Int J Nurs Stud. 2017 Oct;75:43–50.

[65] Klumb PL, Voelkle MC, Siegler S. How negative social interactions at work seep into the home: A prosocial and an antisocial pathway. J Organ Behav. 2017 Oct;38(5):629–649.

[66] Pereira D, Semmer NK, Elfering A. Illegitimate Tasks and Sleep Quality: An Ambulatory Study. Stress Health. 2014 Aug;30(3):209–221.

[67] Stucky ER, Dresselhaus TR, Dollarhide A, Shively M, Maynard G, Jain S, et al. Intern to Attending: Assessing Stress Among Physicians. Acad Med. 2009 Feb;84(2):251–257.

[68] Baethge A, Rigotti T. Interruptions to workflow: Their relationship with irritation and satisfaction with performance, and the mediating roles of time pressure and mental demands. Work Stress. 2013 Jan;27(1):43–63.

[69] Ferdous R, Osmani V, Marquez JB, Mayora O. Investigating correlation between verbal interactions and perceived stress. In: 2015 37th Annual International Conference of the IEEE Engineering in Medicine and Biology Society (EMBC). IEEE; 2015. p. 1612–1615.

[70] Matta FK, Scott BA, Colquitt JA, Koopman J, Passantino LG. Is Consistently Unfair Better than Sporadically Fair? An Investigation of Justice Variability and Stress. Acad Manage J. 2017 Apr;60(2):743–770.

[71] Reicherts M, Pihet S. Job newcomers coping with stressful situations: A microanalysis of adequate coping and well-being. Swiss J Psychol. 2000 Dec;59(4):303–316.

[72] Gervais RL. Menstruation as a Work Stressor: Evidence and Interventions. In: Gervais RL, Millear PM, editors. Exploring Resources, Life-Balance and Well-Being of Women Who Work in a Global Context. Springer International Publishing; 2016. p. 201–218.

[73] Boudreaux E, Jones GN, Mandry C, Brantley PJ. Patient Care and Daily Stress Among Emergency Medical Technicians. Prehospital Disaster Med. 1996 Sep;11(3):188–193.

[74] Shively M, Rutledge T, Rose BA, Graham P, Long R, Stucky E, et al. Real-Time Assessment of Nurse Work Environment and Stress. J Healthc Qual. 2011 Jan;33(1):39–48.

[75] Baethge A, Deci N, Dettmers J, Rigotti T. "Some days won't end ever": Working faster and longer as a boundary condition for challenge versus hindrance effects of time pressure. J Occup Health Psychol. 2019 Jun;24(3):322–332.

[76] Buunk BP, Peeters MCW. Stress at work, social support and companionship: Towards an event-contingent recording approach. Work Stress. 1994 Apr;8(2):177–190.

[77] Weenk M, Alken APB, Engelen LJLPG, Bredie SJH, van de Belt TH, van Goor H. Stress measurement in surgeons and residents using a smart patch. Am J Surg. 2018 Aug;216(2):361–368.

[78] Steptoe A. Stress, social support and cardiovascular activity over the working day. Int J Psychophysiol. 2000 Sep;37(3):299–308.

[79] Johnston D, Bell C, Jones M, Farquharson B, Allan J, Schofield P, et al. Stressors, Appraisal of Stressors, Experienced Stress and Cardiac Response: A Real-Time, Real-Life Investigation of Work Stress in Nurses. Ann Behav Med. 2016 Apr;50(2):187–197.

[80] van Hooff MLM. The Daily Commute from Work to Home: Examining Employees' Experiences in Relation to Their Recovery Status. Stress Health. 2015 Apr;31(2):124–137.

[81] Diebig M, Bormann KC. The dynamic relationship between laissez-faire leadership and day-level stress: A role theory perspective. Ger J Hum Resour Manag. 2020 Jan;34(3):324–344.

[82] Wood S, Michaelides G, Totterdell P. The impact of fluctuating workloads on well-being and the mediating role of work-nonwork interference in this relationship. J Occup Health Psychol. 2013;18(1):106–119.

[83] Levin S, France DJ, Hemphill R, Jones I, Chen KY, Rickard D, et al. Tracking Workload in the Emergency Department. Hum Factors. 2006 Sep;48(3):526–539.

[84] Tadić M, Bakker AB, Oerlemans WGM. Work happiness among teachers: A day reconstruction study on the role of self-concordance. J School Psychol. 2013 Dec;51(6):735–750.

[85] Elfering A, Semmer NK, Grebner S. Work stress and patient safety: Observer-rated work stressors as predictors of characteristics of safety-related events reported by young nurses. Ergonomics. 2006 Apr;49(5-6):457–469.

[86] Almeida DM, Davis KD. Workplace Flexibility and Daily Stress Processes in Hotel Employees and Their Children. Ann Am Acad Politi Soc Sci. 2011 Oct;638(1):123–140.
