# Supplementary material for: Work environment risk factors causing day-to-day stress in occupational settings: a systematic review
Source: BMC Public Health. 2022 Feb 5;22:240. doi: 10.1186/s12889-021-12354-8 (PMC8818147; doi:10.1186/s12889-021-12354-8)
Supplement: Supplementary file 3 — Additional file 3 The full search strategies with indexing terms and free text words for all the databases searched: PubMed, Embase, Web of Science, Scopus, CINAHL, ERIC, and PsycArticles. [file 12889_2021_12354_MOESM3_ESM.pdf]

(“stress”[tiab] OR “stressor”[tiab] OR “distress”[tiab] OR “load”[tiab] OR “workload”[MeSH] OR “workload”[tiab] OR “demand”[tiab] OR “strain”[tiab] OR “pressure”[MeSH] OR “pressure”[tiab])
AND
(“work”[MeSH] OR “worker”[tiab] OR “workers”[tiab] OR “workplace”[MeSH] OR “workplace”[tiab] OR “occupations”[MeSH] OR “occupation”[tiab] OR “occupations”[tiab] OR “profession”[tiab] OR “professions”[tiab] OR “job”[tiab] OR “staff”[tiab] OR “employee”[tiab] OR “employees”[tiab] OR “employment”[MeSH] OR “employment”[tiab] OR “personnel”[tiab])
AND
(

> “ecological momentary assessment”[MeSH] OR “ecological momentary assessment”[tiab] OR “EMA”[tiab] OR “experience sampling”[tiab] OR “ambulatory assessment”[tiab] OR (“intensive”[tiab] AND “longitudinal”[tiab]) OR “interaction record”[tiab] OR “interaction records”[tiab] OR “diary”[tiab] OR “diaries”[tiab] OR “monitoring”[tiab]
> **OR**
> (
>
> > (“continuous”[tiab] OR “repeated”[tiab] OR “multiple”[tiab])
> > **AND**
> > (“measure”[tiab] OR “measures”[tiab] OR “measurement”[tiab] OR “measurements”[tiab] OR “evaluation”[tiab] OR “evaluations”[tiab] OR “assessment”[tiab] OR “assessments”[tiab] OR “checklist”[MeSH] OR “checklist”[tiab] OR “checklists”[tiab] OR “surveys and questionnaires”[MeSH] OR “survey”[tiab] OR “surveys”[tiab] OR “questionnaire”[tiab] OR “questionnaires”[tiab])
>
>)

)
AND
(“real-world”[tiab] OR “real-time”[tiab] OR “everyday”[tiab] OR “daily”[tiab] OR “within-day”[tiab] OR “day-to-day”[tiab] OR “week-by-week”[tiab] OR “periodic”[tiab] OR “episodic”[tiab] OR “acute”[tiab])

Figure 1: The search strategy as used in the PubMed database.

(‘stress’/exp OR ‘stressor’:ab,ti,kw OR ‘distress’:ab,ti,kw OR ‘load’:ab,ti,kw OR ‘workload’/exp OR ‘workload’:ab,ti,kw OR ‘demand’:ab,ti,kw OR ‘strain’:ab,ti,kw OR ‘pressure’/exp OR ‘pressure’:ab,ti,kw)
AND
(‘work’/exp OR ‘worker’:ab,ti,kw OR ‘workers’:ab,ti,kw OR ‘workplace’/exp OR ‘workplace’:ab,ti,kw OR ‘occupation’/exp OR ‘occupation’:ab,ti,kw OR ‘occupations’:ab,ti,kw OR ‘profession’:ab,ti,kw OR ‘professions’:ab,ti,kw OR ‘job’:ab,ti,kw OR ‘staff’/exp OR ‘staff’:ab,ti,kw OR ‘employee’/exp OR ‘employee’:ab,ti,kw OR ‘employees’:ab,ti,kw OR ‘employment’/exp OR ‘employment’:ab,ti,kw OR ‘personnel’/exp OR ‘personnel’:ab,ti,kw)
AND
(

‘ecological momentary assessment’/exp OR ‘ecological momentary assessment’:ab,ti,kw OR ‘EMA’:ab,ti,kw OR ‘experience sampling method’/exp OR ‘experience sampling’:ab,ti,kw OR ‘ambulatory assessment’:ab,ti,kw OR ‘intensive longitudinal’:ab,ti,kw OR ‘interaction record’:ab,ti,kw OR ‘interaction records’:ab,ti,kw OR ‘diary’:ab,ti,kw OR ‘diaries’:ab,ti,kw OR ‘monitoring’/exp OR ‘monitoring’:ab,ti,kw OR ‘computer analysis’/exp
OR
(

(‘continuous’:ab,ti,kw OR ‘repeated’:ab,ti,kw OR ‘multiple’:ab,ti,kw)
AND
(‘measure’:ab,ti,kw OR ‘measures’:ab,ti,kw OR ‘measurement’/exp OR ‘measurement’:ab,ti,kw OR ‘measurements’:ab,ti,kw OR ‘evaluation study’/exp OR ‘evaluation’:ab,ti,kw OR ‘evaluations’:ab,ti,kw OR ‘assessment’/exp OR ‘assessment’:ab,ti,kw OR ‘assessments’:ab,ti,kw OR ‘checklist’/exp OR ‘checklist’:ab,ti,kw OR ‘checklists’:ab,ti,kw OR ‘survey’/exp OR ‘survey’:ab,ti,kw OR ‘surveys’:ab,ti,kw OR ‘questionnaire’/exp OR ‘questionnaire’:ab,ti,kw OR ‘questionnaires’:ab,ti,kw)

)

)
AND
(‘real-world’:ab,ti,kw OR ‘real-time’:ab,ti,kw OR ‘everyday’:ab,ti,kw OR ‘daily’:ab,ti,kw OR ‘within-day’:ab,ti,kw OR ‘day-to-day’:ab,ti,kw OR ‘week-by-week’:ab,ti,kw OR ‘periodic’:ab,ti,kw OR ‘episodic’:ab,ti,kw OR ‘acute’:ab,ti,kw)

Figure 2: The search strategy as used in the Embase database.

(TS=(“stress”) OR TS=(“stressor”) OR TS=(“distress”) OR TS=(“load”) OR TS=(“workload”) OR TS=(“demand”) OR TS=(“strain”) OR TS=(“pressure”))
AND
(TS=(“work”) OR TS=(“worker”) OR TS=(“workers”) OR TS=(“workplace”) OR TS=(“occupation”) OR TS=(“occupations”) OR TS=(“profession”) OR TS=(“professions”) OR TS=(“job”) OR TS=(“staff”) OR TS=(“employee”) OR TS=(“employees”) OR TS=(“employment”) OR TS=(“personnel”))
AND
(

TS=(“ecological momentary assessment”) OR TS=(“EMA”) OR TS=(“experience sampling”) OR TS=(“ambulatory assessment”) OR TS=(“intensive longitudinal”) OR TS=(“interaction record”) OR TS=(“interaction records”) OR TS=(“diary”) OR TS=(“diaries”) OR TS=(“monitoring”)
OR
(

(TS=(“continuous”) OR TS=(“repeated”) OR TS=(“multiple”))
AND
(TS=(“measure”) OR TS=(“measures”) OR TS=(“measurement”) OR TS=(“measurements”) OR TS=(“evaluation”) OR TS=(“evaluations”) OR TS=(“assessment”) OR TS=(“assessments”) OR TS=(“checklist”) OR TS=(“checklists”) OR TS=(“survey”) OR TS=(“surveys”) OR TS=(“questionnaire”) OR TS=(“questionnaires”))

)

)
AND
(TS=(“real-world”) OR TS=(“real-time”) OR TS=(“everyday”) OR TS=(“daily”) OR TS=(“within-day”) OR TS=(“day-to-day”) OR TS=(“week-by-week”) OR TS=(“periodic”) OR TS=(“episodic”) OR TS=(“acute”))

Figure 3: The search strategy as used in the Web Of Science database.

(TITLE-ABS-KEY ({stress}) OR TITLE-ABS-KEY ({stressor}) OR TITLE-ABS-KEY ({distress}) OR TITLE-ABS-KEY ({load}) OR TITLE-ABS-KEY ({workload}) OR TITLE-ABS-KEY ({demand}) OR TITLE-ABS-KEY ({strain}) OR TITLE-ABS-KEY ({pressure}))

AND

(TITLE-ABS-KEY ({work}) OR TITLE-ABS-KEY ({worker}) OR TITLE-ABS-KEY ({workers}) OR TITLE-ABS-KEY ({workplace}) OR TITLE-ABS-KEY ({occupation}) OR TITLE-ABS-KEY ({occupations}) OR TITLE-ABS-KEY ({profession}) OR TITLE-ABS-KEY ({professions}) OR TITLE-ABS-KEY ({job}) OR TITLE-ABS-KEY ({staff}) OR TITLE-ABS-KEY ({employee}) OR TITLE-ABS-KEY ({employees}) OR TITLE-ABS-KEY ({employment}) OR TITLE-ABS-KEY ({personnel}))

AND

(

(TITLE-ABS-KEY ({ecological momentary assessment}) OR TITLE-ABS-KEY ({EMA}) OR TITLE-ABS-KEY ({experience sampling}) OR TITLE-ABS-KEY ({ambulatory assessment}) OR TITLE-ABS-KEY ({intensive longitudinal}) OR TITLE-ABS-KEY ({interaction record}) OR TITLE-ABS-KEY ({interaction records}) OR TITLE-ABS-KEY ({diary}) OR TITLE-ABS-KEY ({diaries}) OR TITLE-ABS-KEY ({monitoring})

OR

(

(TITLE-ABS-KEY ({continuous}) OR TITLE-ABS-KEY ({repeated}) OR TITLE-ABS-KEY ({multiple}))

AND

(TITLE-ABS-KEY ({measure}) OR TITLE-ABS-KEY ({measures}) OR TITLE-ABS-KEY ({measurement}) OR TITLE-ABS-KEY ({measurements}) OR TITLE-ABS-KEY ({evaluation}) OR TITLE-ABS-KEY ({evaluations}) OR TITLE-ABS-KEY ({assessment}) OR TITLE-ABS-KEY ({assessments}) OR TITLE-ABS-KEY ({checklist}) OR TITLE-ABS-KEY ({checklists}) OR TITLE-ABS-KEY ({survey}) OR TITLE-ABS-KEY ({surveys}) OR TITLE-ABS-KEY ({questionnaire}) OR TITLE-ABS-KEY ({questionnaires}))

)

)

AND

(TITLE-ABS-KEY ({real-world}) OR TITLE-ABS-KEY ({real-time}) OR TITLE-ABS-KEY ({everyday}) OR TITLE-ABS-KEY ({daily}) OR TITLE-ABS-KEY ({within-day}) OR TITLE-ABS-KEY ({day-to-day}) OR TITLE-ABS-KEY ({week-by-week}) OR TITLE-ABS-KEY ({periodic}) OR TITLE-ABS-KEY ({episodic}) OR TITLE-ABS-KEY ({acute}))

Figure 4: The search strategy as used in the Scopus database.

(MH “Stress” OR TI “stress” OR AB “stress” OR TI “stressor” OR AB “stressor” OR TI “distress” OR AB “distress” OR TI “load” OR AB “load” OR MH “Workload” OR TI “workload” OR AB “workload” OR TI “demand” OR AB “demand” OR TI “strain” OR AB “strain” OR MH “Pressure” OR TI “pressure” OR AB “pressure”)
AND
(MH “Work” OR TI “worker” OR AB “worker” OR TI “workers” OR AB “workers” OR TI “workplace” OR AB “workplace” OR MH “Occupation (Human)” OR TI “occupation” OR AB “occupation” OR TI “occupations” OR AB “occupations” OR TI “profession” OR AB “profession” OR TI “professions” OR AB “professions” OR TI “job” OR AB “job” OR TI “staff” OR AB “staff” OR TI “employee” OR AB “employee” OR TI “employees” OR AB “employees” OR MH “Employment” OR TI “employment” OR AB “employment” OR TI “personnel” OR AB “personnel”)
AND
(

TI “ecological momentary assessment” OR AB “ecological momentary assessment” OR TI “EMA” OR AB “EMA” OR TI “experience sampling” OR AB “experience sampling” OR TI “ambulatory assessment” OR AB “ambulatory assessment” OR TI “intensive longitudinal” OR AB “intensive longitudinal” OR TI “interaction record” OR AB “interaction record” OR TI “interaction records” OR AB “interaction records” OR MH “Diaries” OR TI “diary” OR AB “diary” OR TI “diaries” OR AB “diaries” OR TI “monitoring” OR AB “monitoring” OR MH “Repeated Measures”
OR
(

(TI “continuous” OR AB “continuous” OR TI “repeated” OR AB “repeated” OR TI “multiple” OR AB “multiple”)
AND
(TI “measure” OR AB “measure” OR TI “measures” OR AB “measures” OR TI “measurement” OR AB “measurement” OR TI “measurements” OR AB “measurements” OR MH “Evaluation” OR TI “evaluation” OR AB “evaluation” OR TI “evaluations” OR AB “evaluations” OR TI “assessment” OR AB “assessment” OR TI “assessments” OR AB “assessments” OR MH “Checklists” OR TI “checklist” OR AB “checklist” OR TI “checklists” OR AB “checklists” OR MH “Surveys” OR TI “survey” OR AB “survey” OR TI “surveys” OR AB “surveys” OR MH “Questionnaires” OR TI “questionnaire” OR AB “questionnaire” OR TI “questionnaires” OR AB “questionnaires”)

)

)
AND
(TI “real-world” OR AB “real-world” OR TI “real-time” OR AB “real-time” OR TI “everyday” OR AB “everyday” OR TI “daily” OR AB “daily” OR TI “within-day” OR AB “within-day” OR TI “day-to-day” OR AB “day-to-day” OR TI “week-by-week” OR AB “week-by-week” OR TI “periodic” OR AB “periodic” OR TI “episodic” OR AB “episodic” OR TI “acute” OR AB “acute”)

Figure 5: The search strategy as used in the CINAHL database.

(ab(“stress”) OR ti(“stress”) OR if(“stress”) OR ab(“stressor”) OR ti(“stressor”) OR if(“stressor”) OR ab(“distress”) OR ti(“distress”) OR if(“distress”) OR ab(“load”) OR ti(“load”) OR if(“load”) OR MAINSUBJECT.EXACT(“Teaching Load”) OR ab(“workload”) OR ti(“workload”) OR if(“workload”) OR ab(“demand”) OR ti(“demand”) OR if(“demand”) OR ab(“strain”) OR ti(“strain”) OR if(“strain”) OR ab(“pressure”) OR ti(“pressure”) OR if(“pressure”))

AND

(ab(“work”) OR ti(“work”) OR if(“work”) OR ab(“worker”) OR ti(“worker”) OR if(“worker”) OR ab(“workers”) OR ti(“workers”) OR if(“workers”) OR ab(“workplace”) OR ti(“workplace”) OR if(“workplace”) OR ab(“occupation”) OR ti(“occupation”) OR if(“occupation”) OR ab(“occupations”) OR ti(“occupations”) OR if(“occupations”) OR ab(“profession”) OR ti(“profession”) OR if(“profession”) OR ab(“professions”) OR ti(“professions”) OR if(“professions”) OR ab(“job”) OR ti(“job”) OR if(“job”) OR ab(“staff”) OR ti(“staff”) OR if(“staff”) OR MAINSUBJECT.EXACT(“Employees”) OR ab(“employee”) OR ti(“employee”) OR if(“employee”) OR ab(“employees”) OR ti(“employees”) OR if(“employees”) OR MAINSUBJECT.EXACT(“Employment”) OR ab(“employment”) OR ti(“employment”) OR if(“employment”) OR ab(“personnel”) OR ti(“personnel”) OR if(“personnel”))

AND

(

ab(“ecological momentary assessment”) OR ti(“ecological momentary assessment”) OR if(“ecological momentary assessment”) OR ab(“EMA”) OR ti(“EMA”) OR if(“EMA”) OR ab(“experience sampling”) OR ti(“experience sampling”) OR if(“experience sampling”) OR ab(“ambulatory assessment”) OR ti(“ambulatory assessment”) OR if(“ambulatory assessment”) OR ab(“intensive longitudinal”) OR ti(“intensive longitudinal”) OR if(“intensive longitudinal”) OR ab(“interaction record”) OR ti(“interaction record”) OR if(“interaction record”) OR ab(“interaction records”) OR ti(“interaction records”) OR if(“interaction records”) OR MAINSUBJECT.EXACT(“Diaries”) OR ab(“diary”) OR ti(“diary”) OR if(“diary”) OR ab(“diaries”) OR ti(“diaries”) OR if(“diaries”) OR ab(“monitoring”) OR ti(“monitoring”) OR if(“monitoring”)

OR

(

(ab(“continuous”) OR ti(“continuous”) OR if(“continuous”) OR ab(“repeated”) OR ti(“repeated”) OR if(“repeated”) OR ab(“multiple”) OR ti(“multiple”) OR if(“multiple”))

AND

(ab(“measure”) OR ti(“measure”) OR if(“measure”) OR ab(“measures”) OR ti(“measures”) OR if(“measures”) OR MAINSUBJECT.EXACT(“Measurement”) OR ab(“measurement”) OR ti(“measurement”) OR if(“measurement”) OR ab(“measurements”) OR ti(“measurements”) OR if(“measurements”) OR MAINSUBJECT.EXACT(“Evaluation”) OR MAINSUBJECT.EXACT(“Evaluation Methods”) OR ab(“evaluation”) OR ti(“evaluation”) OR if(“evaluation”) OR ab(“evaluations”) OR ti(“evaluations”) OR if(“evaluations”) OR ab(“assessment”) OR ti(“assessment”) OR if(“assessment”) OR ab(“assessments”) OR ti(“assessments”) OR if(“assessments”) OR MAINSUBJECT.EXACT(“Check Lists”) OR ab(“checklist”) OR ti(“checklist”) OR if(“checklist”) OR ab(“checklists”) OR ti(“checklists”) OR if(“checklists”) OR MAINSUBJECT.EXACT(“Surveys”) OR ab(“survey”) OR ti(“survey”) OR if(“survey”) OR ab(“surveys”) OR ti(“surveys”) OR if(“surveys”) OR MAINSUBJECT.EXACT(“Questionnaires”) OR ab(“questionnaire”) OR ti(“questionnaire”) OR if(“questionnaire”) OR ab(“questionnaires”) OR ti(“questionnaires”) OR if(“questionnaires”))

)

)

AND

(ab(“real-world”) OR ti(“real-world”) OR if(“real-world”) OR ab(“real-time”) OR ti(“real-time”) OR if(“real-time”) OR ab(“everyday”) OR ti(“everyday”) OR if(“everyday”) OR ab(“daily”) OR ti(“daily”) OR if(“daily”) OR ab(“within-day”) OR ti(“within-day”) OR if(“within-day”) OR ab(“day-to-day”) OR ti(“day-to-day”) OR if(“day-to-day”) OR ab(“week-by-week”) OR ti(“week-by-week”) OR if(“week-by-week”) OR ab(“periodic”) OR ti(“periodic”) OR if(“periodic”) OR ab(“episodic”) OR ti(“episodic”) OR if(“episodic”) OR ab(“acute”) OR ti(“acute”) OR if(“acute”))

Figure 6: The search strategy as used in the ERIC database.

(MAINSUBJECT.EXACT(“Stress”) OR ab(“stress”) OR ti(“stress”) OR if(“stress”) OR ab(“stressor”) OR ti(“stressor”) OR if(“stressor”) OR MAINSUBJECT.EXACT(“Occupational Stress”) OR MAINSUBJECT.EXACT(“Distress”) OR ab(“distress”) OR ti(“distress”) OR if(“distress”) OR ab(“load”) OR ti(“load”) OR if(“load”) OR MAINSUBJECT.EXACT(“Work Load”) OR ab(“workload”) OR ti(“workload”) OR if(“workload”) OR ab(“demand”) OR ti(“demand”) OR if(“demand”) OR ab(“strain”) OR ti(“strain”) OR if(“strain”) OR ab(“pressure”) OR ti(“pressure”) OR if(“pressure”))
AND
(ab(“work”) OR ti(“work”) OR if(“work”) OR ab(“worker”) OR ti(“worker”) OR if(“worker”) OR ab(“workers”) OR ti(“workers”) OR if(“workers”) OR ab(“workplace”) OR ti(“workplace”) OR if(“workplace”) OR MAINSUBJECT.EXACT(“Occupations”) OR ab(“occupation”) OR ti(“occupation”) OR if(“occupation”) OR ab(“occupations”) OR ti(“occupations”) OR if(“occupations”) OR ab(“profession”) OR ti(“profession”) OR if(“profession”) ab(“professions”) OR ti(“professions”) OR if(“professions”) OR ab(“job”) OR ti(“job”) OR if(“job”) OR ab(“staff”) OR ti(“staff”) OR if(“staff”) OR ab(“employee”) OR ti(“employee”) OR if(“employee”) OR ab(“employees”) OR ti(“employees”) OR if(“employees”) OR ab(“employment”) OR ti(“employment”) OR if(“employment”) OR MAINSUBJECT.EXACT(“Personnel”) OR ab(“personnel”) OR ti(“personnel”) OR if(personnel))
AND
(

ab(“ecological momentary assessment”) OR ti(“ecological momentary assessment”) OR if(“ecological momentary assessment”) OR ab(“EMA”) OR ti(“EMA”) OR if(“EMA”) OR ab(“experience sampling”) OR ti(“experience sampling”) OR if(“experience sampling”) OR ab(“ambulatory assessment”) OR ti(“ambulatory assessment”) OR if(“ambulatory assessment”) OR ab(“intensive longitudinal”) OR ti(“intensive longitudinal”) OR if(“intensive longitudinal”) OR ab(“interaction record”) or ti(“interaction record”) OR if(“interaction record”) OR ab(“interaction records”) or ti(“interaction records”) OR if(“interaction records”) OR MAINSUBJECT.EXACT(“Journal Writing”) OR ab(“diary”) OR ti(“diary”) OR if(“diary”) OR ab(“diaries”) OR ti(“diaries”) OR if(“diaries”) OR MAINSUBJECT.EXACT(“Monitoring”) OR ab(“monitoring”) OR ti(“monitoring”) OR if(“monitoring”) OR MAINSUBJECT.EXACT(“Repeated Measures”)

OR

(

(ab(“continuous”) OR ti(“continuous”) OR if(“continuous”) OR ab(“repeated”) OR ti(“repeated”) OR if(“repeated”) OR ab(“multiple”) OR ti(“multiple”) OR if(“multiple”))

AND

(ab(“measure”) OR ti(“measure”) OR if(“measure”) OR ab(“measures”) OR ti(“measures”) OR if(“measures”) OR MAINSUBJECT.EXACT(“Measurement”) OR ab(“measurement”) OR ti(“measurement”) OR if(“measurement”) OR ab(“measurements”) OR ti(“measurements”) OR if(“measurements”) OR MAINSUBJECT.EXACT(“Evaluation”) OR ab(“evaluation”) OR ti(“evaluation”) OR if(“evaluation”) OR ab(“evaluations”) OR ti(“evaluations”) OR if(“evaluations”) OR ab(“assessment”) OR ti(“assessment”) OR if(“assessment”) OR ab(“assessments”) OR ti(“assessments”) OR if(“assessments”) OR MAINSUBJECT.EXACT(“Checklist (Testing)”) OR ab(“checklist”) OR ti(“checklist”) OR if(“checklist”) OR ab(“checklists”) OR ti(“checklists”) OR if(“checklists”) OR MAINSUBJECT.EXACT(“Surveys”) OR ab(“survey”) OR ti(“survey”) OR if(“survey”) OR ab(“surveys”) OR ti(“surveys”) OR if(“surveys”) OR MAINSUBJECT.EXACT(“Questionnaires”) OR ab(“questionnaire”) OR ti(“questionnaire”) OR if(“questionnaire”) OR ab(“questionnaires”) OR ti(“questionnaires”) OR if(“questionnaires”))

)

)
AND

(ab(“real-world”) OR ti(“real-world”) OR if(“real-world”) OR ab(“real-time”) OR ti(“real-time”) OR if(“real-time”) OR ab(“everyday”) OR ti(“everyday”) OR if(“everyday”) OR ab(“daily”) OR ti(“daily”) OR if(“daily”) OR ab(“within-day”) OR ti(“within-day”) OR if(“within-day”) OR ab(“day-to-day”) OR ti(“day-to-day”) OR if(“day-to-day”) OR ab(“week-by-week”) OR ti(“week-by-week”) OR if(“week-by-week”) OR ab(“periodic”) OR ti(“periodic”) OR if(“periodic”) OR ab(“episodic”) OR ti(“episodic”) OR if(“episodic”) OR ab(“acute”) OR ti(“acute”) OR if(“acute”))

Figure 7: The search strategy as used in the PsycArticles database.
